# Methodological limitations in experimental studies on symptom development in individuals with idiopathic environmental intolerance attributed to electromagnetic fields (IEI-EMF) – a systematic review

**DOI:** 10.1186/s12940-019-0519-x

**Published:** 2019-10-22

**Authors:** Kristina Schmiedchen, Sarah Driessen, Gunnhild Oftedal

**Affiliations:** 1 Research Center for Bioelectromagnetic Interaction, Institute for Occupational, Social and Environmental Medicine, RWTH Aachen University, Pauwelsstraße 30, 52074 Aachen, Germany; 2 Department of Electronic Systems, Faculty of Information Technology and Electrical Engineering, Norwegian University of Science and Technology – NTNU, Trondheim, Norway

**Keywords:** Electromagnetic field, Idiopathic environmental intolerance, Electromagnetic hypersensitivity, Symptom, Provocation study, Methodological limitation

## Abstract

**Background:**

Hypersensitivity to electromagnetic fields (EMF) is a controversial condition. While individuals with idiopathic environmental intolerance attributed to electromagnetic fields (IEI-EMF) claim to experience health complaints upon EMF exposure, many experimental studies have found no convincing evidence for a physical relation. The aim of this systematic review was to evaluate methodological limitations in experimental studies on symptom development in IEI-EMF individuals that might have fostered false positive or false negative results. Furthermore, we compared the profiles of these limitations between studies with positive and negative results.

**Methods:**

The Preferred Reporting Items for Systematic Reviews and Meta-Analyses (PRISMA) guided the methodological conduct and reporting. Eligible were blinded experimental studies that exposed individuals with IEI-EMF to different EMF exposure levels and queried the development of symptoms during or after each exposure trial. Strengths and limitations in design, conduct and analysis of individual studies were assessed using a customized rating tool.

**Results:**

Twenty-eight studies met the eligibility criteria and were included in this review. In many studies, both with positive and negative results, we identified methodological limitations that might have either fostered false or masked real effects of exposure. The most common limitations were related to the selection of study participants, the counterbalancing of the exposure sequence and the effectiveness of blinding. Many studies further lacked statistical power estimates. Methodically sound studies indicated that an effect of exposure is unlikely.

**Conclusion:**

Overall, the evidence points towards no effect of exposure. If physical effects exist, previous findings suggest that they must be very weak or affect only few individuals with IEI-EMF. Given the evidence that the nocebo effect or medical/mental disorders may explain the symptoms in many individuals with IEI-EMF, additional research is required to identify the various factors that may be important for developing IEI-EMF and for provoking the symptoms. We recommend the identification of subgroups and exploring IEI-EMF in the context of other idiopathic environmental intolerances. If further experimental studies are conducted, they should preferably be performed at the individual level. In particular, to increase the likelihood of detecting hypersensitive individuals, if they exist, we encourage researchers to achieve a high credibility of the results by minimizing sources of risk of bias and imprecision.

**Electronic supplementary material:**

The online version of this article (10.1186/s12940-019-0519-x) contains supplementary material, which is available to authorized users.

## Introduction

Idiopathic environmental intolerance attributed to electromagnetic fields (IEI-EMF) – more commonly known as electromagnetic hypersensitivity (EHS) – is still a matter of scientific debate and much of the controversy has centred on the question of whether the condition is truly caused by electromagnetic fields (EMF). In contrast to most people, the afflicted individuals claim to suffer from health complaints when using or being in the vicinity of EMF exposure sources, therefore using terms like hypersensitivity or intolerance to EMF to describe their condition [1–4]. Because the aetiology of this condition remains unknown, the term IEI-EMF is often used to describe that medically unexplained symptoms are attributed to EMF. Individuals with IEI-EMF typically complain about non-specific symptoms such as headaches, fatigue, sleep disturbances, nausea, lack of concentration, skin irritation and muscle pain [1, 5–10]. Exposure sources that are reported to cause the symptoms include mobile phones, WiFi routers, visual display units (VDU), microwaves, base stations, high-voltage power lines, and radars [1, 6, 8]. Some of the individuals with IEI-EMF severely suffer from impaired health status and feel restricted in daily life and in their performance of normal routines [3, 8, 11, 12].

Experimental provocation studies in which participants are exposed to active (EMF) and inactive (sham) conditions have been conducted to examine whether EMF can cause the symptoms. However, there is currently no scientifically sound evidence supporting a causal relation between exposure to EMF and health problems. After assessment of the research findings, the World Health Organization (WHO) [4] (fact sheet No. 296) and the European Scientific Committee on Emerging and Newly Identified Health Risks (SCENIHR) [13] considered it unlikely that short-term exposure to EMF can trigger non-specific symptoms. Also, several systematic reviews that evaluated the results of experimental studies testing the effect of exposure on symptom development and well-being (subjective outcomes) [14–18] and/or on physiological/cognitive parameters (objective outcomes) [14, 16, 19] in individuals suffering from IEI-EMF came to the same conclusion. It was therefore proposed that factors unrelated to EMF underlie the development of symptoms in individuals with IEI-EMF [14, 15, 18]. Several findings support the role of the nocebo effect [20–24], i.e., the mere belief about the harmful effects of EMF may provoke symptoms in some individuals, and such negative expectations may partly be fostered by media reports [25–27]. Other studies found evidence that misattribution [28–30], severe medical and social problems [31, 32], an imbalance in the autonomic nervous system [33–35], or psychosomatic disorders [8, 9, 31, 36] may play a role in causing the symptoms.

Studies suggesting a physical relation between EMF exposure and health complaints have been criticized for methodological limitations including inadequate counterbalancing of the exposure sequence, inappropriate blinding of participants or missing adjustment for multiple comparisons [18]. These limitations might have resulted in false positive results, i.e., the results indicate an effect that actually is not present. However, experimental studies on IEI-EMF indicating no effect of exposure may also have been influenced by methodological limitations that might explain why they did not find an effect, if a relation exists. Limitations related to the experimental procedure or to the procedure to select participants might have masked effects of exposure and fostered false negative results, i.e., the results indicate that there is no effect when it is actually present. Several authors noted that among individuals with IEI-EMF, only a small subgroup might exist whose symptoms are caused by physical effects of EMF exposure [15, 18, 29, 37–41] and their responses could be masked in heterogeneous study groups that include individuals misattributing symptoms provoked by e.g. somatic diseases or mental disorders to EMF exposure [18]. Some authors therefore recommended the careful assessment of differences between subgroups [19, 41–44], but this would only be possible if the studies gathered sufficient data about their participants.

The aim of this systematic review was to evaluate methodological limitations that might have fostered false positive or false negative results in experimental studies examining the relation between EMF exposure and symptoms reported by individuals with IEI-EMF. A particular goal was to fill a gap within the literature on IEI-EMF: previously published systematic reviews of experimental studies with subjective outcomes either considered exposure sources within a limited frequency range [15, 16] and/or did not assess the methodological quality of individual studies [14, 15, 17, 18]. A risk-of-bias assessment was only provided by Röösli et al. [16] in a systematic review of studies using exposure sources in the RF range. However, no comprehensive assessment of the methodological quality of experimental studies with subjective outcomes has been published so far for the various EMF exposure sources in the frequency range of 0–300 GHz. We did not include objective outcomes in our analysis because symptoms and reduced well-being are the primary outcomes associated with IEI-EMF and are more relevant for individuals with IEI-EMF based on their complaints. A systematic review of physiological effects in individuals with IEI-EMF, including a comprehensive assessment of the methodological quality of individual studies has been provided by Rubin et al. [19]. Furthermore, the experimental research designs differ between studies investigating symptom development and those investigating physiological and cognitive functioning which precludes their joint analysis in this review.

We evaluated the included studies by applying a customized rating tool consisting of 16 key questions to identify strengths and limitations in design, conduct and analysis of individual studies. The identified limitations might have given rise to bias or imprecision. We assessed for example limitations regarding the selection of participants, the sequence generation, the control of exposures or the blinding of participants and research personal. To each key question related to risk of bias, we assigned a direction of bias it would have on the study outcome. Furthermore, we compared the profiles of limitations between studies with positive results (statistically significant outcomes) and studies with negative results (non-statistically significant outcomes). This review will contribute to assessing the credibility of the outcomes of previous experimental studies and to identifying research needs and priorities in IEI-EMF research.

## Methods

The Preferred Reporting Items for Systematic Reviews and Meta-Analyses (PRISMA) [45] was used to guide the methodological conduct and the reporting of this systematic review. The search strategy, the inclusion and exclusion criteria and the data to be extracted from eligible articles were pre-specified in a protocol before the search for relevant articles. Prior to data extraction, we developed a customized rating tool to assess the methodological quality of eligible studies (see Risk of bias and imprecision assessment). A few amendments were made post hoc to the rating tool by adding less common methodological approaches that we identified during the extraction procedure. Not part of the protocol before data extraction were (1) a revisited rating of the methodological quality of the studies and (2) the statistical comparisons between studies with positive and negative results regarding their profiles of limitations.

### Eligibility criteria

The eligibility criteria were defined using the Participant, Intervention, Control, Outcome, and Study design (PICOS) criteria [46]. Peer-reviewed journal articles written in English and German were eligible for this review if they described experimental provocation or intervention studies (S) with individuals suffering from IEI-EMF (P). The primary inclusion criterion was that studies examined the well-being or the number/severity of symptoms (O) upon exposure to EMF in the frequency range between 0 and 300 GHz (I). Studies were only eligible if they exposed participants to at least two conditions with different exposure levels (C) but otherwise identical experimental parameters and queried the symptoms during or after each individual exposure trial to allow for comparison between the exposure conditions. We only considered studies in which the exposures were blinded to participants (single-blind) or blinded to participants and the research personal (double-blind). We excluded observational epidemiological studies and studies that examined alterations of physiological or cognitive parameters or the effect of therapeutic approaches. Also excluded were reviews, conference proceedings, letters to the editor, comments, guidelines for practitioners, or articles that described the design and conduct of a planned study. No restrictions were applied as to the year of publication.

### Information sources and literature search

Relevant articles published through March 2019 were identified through electronic database searches in PubMed (U.S. National Library of Medicine, National Institutes of Health), Web of Science (Institute for Scientific Information, Clarivate Analytics), Cochrane Library (Cochrane, John Wiley & Sons), PsychInfo (American Psychological Association, APA PsycNET) and the EMF-Portal (Research Center for Bioelectromagnetic Interaction, RWTH Aachen University). Search terms were related to participants (e.g., IEI-EMF, EHS, electromagnetic hypersensitivity, environmental intolerance), exposures (e.g., electromagnetic, mobile phone, power line, GSM, visual display unit) and study outcomes (e.g., well-being, ill health, symptom, health complaint). These terms were always combined to limit the identified articles to those that were relevant to the topic. The search strings and links to the electronic databases are provided in the Additional file 1. To supplement the electronic database searches, we identified additional records through checking reference lists of the retrieved journal articles and reviews.

### Study selection

In a first stage of assessment, the titles and abstracts of the identified and potentially relevant articles were independently screened and assessed by two authors (KS, SD). Duplicate articles and articles which failed to meet the inclusion criteria were sorted out. In the second stage of assessment, the full texts of the potentially eligible articles were obtained and independently reviewed by two authors (KS, SD). The two authors then jointly made a final decision about the inclusion of the articles.

### Data extraction

Two authors (KS, SD) independently extracted details relating to study design, conduct and analysis. The data were jointly compiled. The third author (GO) rechecked the extracted data and in instances where disagreements occurred between the authors, they were discussed and uncertainties were solved by consensus between the three authors.

The extracted data included:

(1) the sample size, (2) the criteria applied to exclude individuals whose EMF-attributed symptoms may be explained by somatic diseases or mental disorders, (3) the method used to identify exposure sources and situations that are associated with the symptoms, (4) the method used to identify the types of symptoms experienced in everyday life, (5) the method used to verify the contrast in severity of symptoms between situations with and without exposure, (6) the method used to assess how quickly symptoms appear and how long they last, (7) the type of exposure source, the frequency range and the exposure level used in the experimental sessions and the duration of exposures, (8) the interval between two consecutive exposure trials, (9) the number of repetitions of each exposure condition, (10) the types of recorded symptoms and the tools used to record the symptoms in the experimental sessions, (11) the assessment times for querying the symptoms in the experimental sessions, (12) the method and level of blinding, (13) the methods used to minimize biases related to the sequence and to the period of the exposure conditions (e.g., randomization, counterbalancing), (14) the methods used to control for co-variates that might bias the outcome, (15) the method used to control and minimize the background exposure level, (16) the method used to control the emission level from the exposure source and/or the exposure level, (17) the level of completeness of the data that were included in the analysis, (18) the number of participants that withdrew from the study and at which stages, (19) the results reported, (20) the level of completeness of the reported outcomes, (21) the statistical power estimates, and (22) the method applied to adjust for multiple comparisons when relevant.

### Risk of bias and imprecision assessment

The extracted data provided the basis for assessing the methodological quality of the included studies in terms of risk of bias and imprecision. Bias refers to a systematic error or deviation from the truth, in results or inferences that may lead to an over- or underestimation of an effect, while imprecision refers to a random error due to too small sample sizes or too low numbers of events [47].

The rating tool customized to experimental studies on symptom development in individuals with IEI-EMF consisted of 16 key questions (risk of bias: 14 key questions, imprecision: 2 key questions). The development of this tool was based on the guidance from the Cochrane Collaboration’s tools [47] and the Grading of Recommendations Assessment, Development and Evaluation (GRADE) approach [48]. For each key question, several methodological alternatives were specified of which at least one alternative was judged appropriate to reduce the likelihood for false positive or false negative results, and at least one alternative (except for one key question, see below) was judged inappropriate and can be considered a source of high risk of bias or imprecision (mainly labelled as “not reported”, meaning that none of the other alternatives were applied or reported in the paper, see Additional file 1: Table S1) To each key question related to risk of bias, we assigned a direction of bias it would have on the study outcome: in favour of an effect of exposure (+), in favour of a null result (−), or uncertain direction on the study outcome, i.e., in favour of either an effect of exposure or a null result (±). Table 1 summarizes the key questions according to the various directions of bias. In Additional file 1: Table S1 and in the relevant figures**,** the direction of bias is indicated by signs (+, − and ±). Note that only for the key question related to level and method of blinding any bias would be in favour of an effect of exposure, while for seven key questions any bias would be in favour of a null result, and for five key questions any bias would have an uncertain direction on the study outcome. Based on this rating tool, we identified strengths and limitations in design, conduct and analysis of individual studies by assessing which of the alternatives under each key question were applied or were relevant for the study.

**Table 1:** Direction of bias on study outcome for each key question

Bias direction	Within domain/key question	Methodological alternatives considered a source of high risk of bias
In favour of an effect of exposure (+)	Performance bias: - Was the level and method of blinding appropriate?	- no blinding of research personal during sessions - insufficient removal of any clues that could reveal exposure status and no tests done to control blinding
In favour of a null result (−)	Selection bias:	
	- Were individuals excluded whose symptoms may be explained by somatic diseases or mental disorders?	- not sufficiently considered/not reported
	- Was the contrast in the severity of symptoms between situations with/without exposure verified?	- not reported
	- Were EMF exposures (type of exposure source, frequency range and exposure level) applied that individuals associate with their symptoms?	- not reported
	- Were exposure durations and assessment times applied that matched the time scales for the symptoms to appear?	- not reported
	- Were the symptoms registered in the trials matched with those experienced in everyday exposure situations?	- not reported
	Exposure bias:	
	- Was the background exposure level controlled and minimized?	- not reported
	- Was the exposure level controlled?	- not reported
Uncertain direction on study outcome (±)	Selection bias:	
	- Were the intervals between exposure sessions sufficiently long to allow for recovery and to avoid carry-over effects?	- not reported
	Performance bias:	
	- Were biases related to sequence and period of the exposure conditions minimized (for studies with cross-over design)?	- same sequence and period of the exposure conditions for all participants or for all participants of a group - not reported
	Confounding bias:	
	- Were biases related to confounders and cofactors minimized (for studies comparing parallel groups of IEI-EMF participants with different exposure conditions)?	- not randomized
	Attrition bias:	
	- Were biases minimized that are related to attrition and to incomplete data included in the analysis?	- high attrition/exclusion rate or incomplete data in analysis
	Selective reporting bias:	
	- Was bias related to selective outcome reporting minimized?	- selective outcome reporting

The 14 key questions related to risk of bias were grouped into six domains: selection of study participants, performance, confounding, detection, attrition, and selective reporting. Customized to experimental studies with IEI-EMF individuals were in particular the aspects considered in the domains selection bias and performance bias. Under *selection bias,* we assessed whether the study included individuals with somatic diseases or mental disorders that may explain their EMF-attributed symptoms and whether the study design was appropriate for the included participants regarding e.g. the symptoms being recorded or the exposures being applied. Under *performance bias*, we considered biases related to knowledge of which exposure condition was used and biases related to the sequence (which may be due to carry-over effects) and to the period of the exposure conditions (which may be due to habituation or variable stress levels as a function of time). The specified co-variates relevant for the key question “Were other co-variates appropriately controlled” under *confounding bias* are not related to the exposure condition but may influence the outcomes (e.g., use of an adaptation period, inclusion of pre-trial symptom levels in the analysis, or control of temperature, humidity and light). Therefore, in randomized trials that did not control any of these co-variates, we did not consider this to cause a high risk of bias. If the sequence of exposure was not randomized, this was considered a source of high risk for bias and addressed under *performance bias*. *Exposure bias* considered potential biases introduced by the use of inappropriate methods to control or assess exposures, including the background exposure. *Attrition bias* was concerned with biases due to withdrawals from the study or incomplete data included in the analysis. Under *selective reporting bias,* we assessed to what extent relevant outcomes related to symptom scores or symptom levels were incomplete.

One domain was defined for *imprecisio*n and included two key questions. The first question is related to concerns regarding statistical power. Sufficient statistical power can be demonstrated with power estimates or a high number of participants or repetitions of trials. When the power to detect an effect of exposure is low due to too few participants or trials, the effect estimates will be imprecise. In addition to a lack of demonstration of sufficient statistical power, we considered the power to be too low when the conclusions were based on descriptive statistics only. The second key question is related to concern regarding missing adjustment for multiple comparisons when relevant for a study. Missing adjustment would increase the likelihood for false positive results. Adjustment was regarded as not relevant (N/A) for studies that conducted no more than two statistical tests (e.g., examined one or two symptoms or the analyses were based on a total symptom score), or for studies that did not provide any statistical analysis. Studies explicitly pre-defining a primary effect variable (i.e., one main symptom while other symptoms were secondary or explorative) were rated like studies that examined several symptoms because in this review we regarded any statistically significant result, including secondary outcomes, to be a positive result. A more detailed description of the various methodological alternatives and the criteria for judging the 16 key questions is provided in the Additional file 1: Table S1).

### Statistical analysis

The results of the risk-of-bias and imprecision assessment of the included studies were used to test whether studies with positive and negative results (independent variables) differed regarding the distribution of key questions judged to be at high risk of bias or judged to have concern regarding precision (dependent variables). Four dependent variables were specified for risk of bias and two for imprecision. For statistical comparisons in which we included a dependent variable with a binary outcome (i.e., based on one key question that was either appropriately addressed or judged to be at high risk or bias/ judged to have concern regarding precision) we used Chi-square test. This test was relevant for the analysis of three key questions and the dependent variables were (i) the number of studies judged to be at high risk of bias in favour of an effect of exposure (+), (ii) the number of studies judged to have concern regarding statistical power and (iii) the number of studies judged to have concern regarding missing adjustment for multiple comparisons. For the remaining comparisons, Student’s t-test would have been applied if the assumptions of this parametric test had been fulfilled, which was not the case: all data distributions differed statistically significantly from normality (Kolmogorov-Smirnov test) and one of the sample sizes was low. Therefore, Mann-Whitney-U-test was used. The three dependent variables were (i) the total number of key questions per study judged to be at high risk of bias, (ii) the number of key questions per study judged to be at high risk of bias in favour of a null result (−) and (iii) the number of key questions per study judged to be at high risk of bias with an uncertain direction on the study outcome (±). All statistical comparisons were done two-tailed. For the analysis of the risk of bias assessment, we regarded the statistical test for the total number of key questions per study judged to be at high risk of bias as the primary test, with the significance level set at α = 0.05. The three tests regarding the direction of bias on study outcome were considered as secondary tests and Bonferroni adjustment of significance levels was applied (α = 0.017). Because two independent statistical tests were performed for imprecision, the significance levels were adjusted accordingly (α = 0.025). SPSS version 19 statistics were applied.

To our knowledge, no similar review study has been published before. Therefore, we estimated the statistical power of our performed analyses partly based on parameters from the current dataset (the distribution of the total number of key questions judged to be at high risk of bias) as suggested by Dziak et al. [49]. The power estimate was calculated using the *ClinCalc* online tool [50], by selecting the options “Two independent study groups” and by assuming Student’s t-test, i.e., “Continuous (means)” was selected as primary endpoint. For the comparison of the two groups of studies (positive and negative results), we considered a difference in means of μ_d_ = 2 in the number of key questions judged to be at high risk of bias to be informative as to whether the dependent variables (e.g., the direction of bias) are crucial factors for study outcomes. By using as sample sizes the number of reviewed studies in each of the two groups of studies and a standard deviation of σ = 2.5 which was representative for the distribution of the total number of key questions judged to be at high risk of bias, the estimated power was 0.45. A power estimate of 0.8 or greater is commonly regarded as sufficient to detect a true effect. Note that the statistical power for the applied Mann-Whitney-U-tests would be somewhat lower than this estimate which is based on Student’s t-test [51].

## Results

### Study selection

**Fig. 1:**
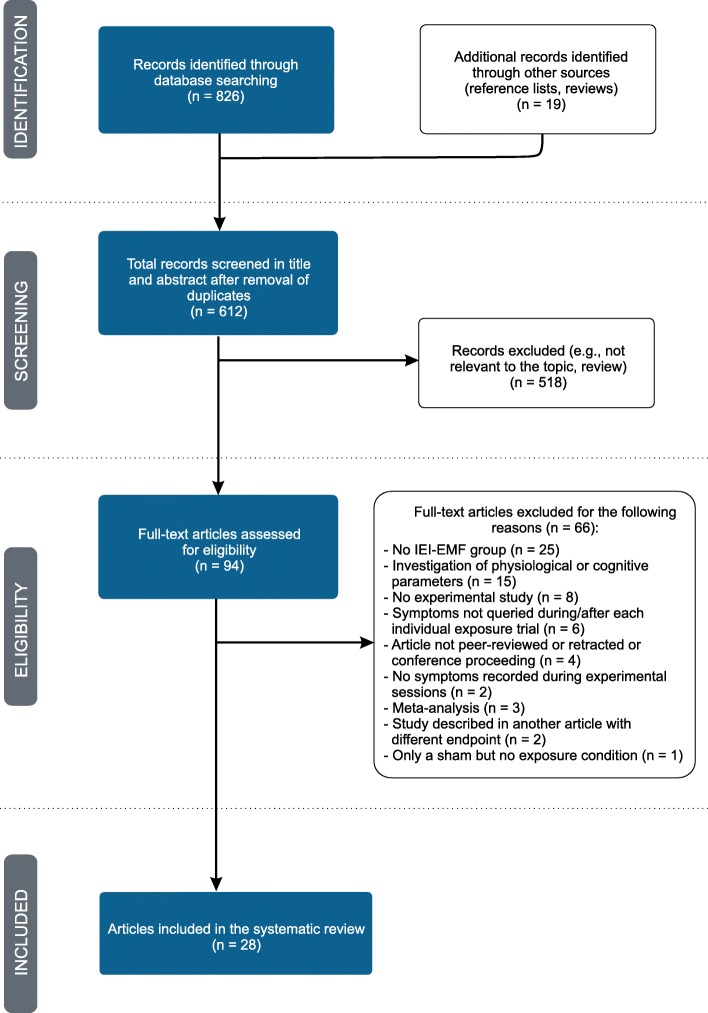
Flow diagram of literature search, eligibility and inclusion process. Adapted from Moher et al. [45]

The systematic search returned a total of 845 articles. After removal of duplicates and exclusion of studies which did not match the eligibility criteria, 28 articles were selected and included in this review (see Fig. 1 for details). A total of 1540 participants were tested in these 28 studies of which 747 (49%) were IEI-EMF individuals and 793 (51%) were controls.

### Applied exposures and effects of exposure

Of the included articles, 26 reported experimental provocation studies and two reported intervention studies where interventions in the work environment reduced the exposure level. Six studies used EMF from a VDU, six used extremely low frequency (ELF) electric or magnetic fields, 15 used RF-EMF and one used magnetic fields (MF) of varying frequencies between 0.1 Hz and 5 MHz.

Effects of exposure on individuals with IEI-EMF were reported in seven studies. Of these seven studies, four reported more severe or a higher number of symptoms [52–55] and three found less severe or a lower number of symptoms [56–58]. Furthermore, five of these seven studies tested only IEI-EMF individuals or found an indication of an effect of exposure only in the group of IEI-EMF individuals [52–54, 57, 58], while two studies found altered symptom levels in the combined group of individuals with IEI-EMF and healthy controls [55, 56]. Beyond these seven studies, Hillert et al. [39] reported that only the healthy control group showed reactions to RF signals. In the following analyses, however, we do not consider results that were relevant only for healthy individuals. Thus, 21 studies did not find evidence that the symptoms reported by IEI-EMF individuals were related to EMF exposures. Studies suggesting an effect of exposure tested 245 (33%) of the IEI-EMF individuals while studies reporting no effect of exposure tested 502 (67%) of the IEI-EMF individuals. Individual study characteristics including a brief description of the results are summarized in Table 2.

**Table 2:** Characteristics of individual studies. The studies are grouped according to type of exposure or frequency range applied. Details on the results are given for statistically significant results. “No statistically significant effect of exposure” indicates that the development of symptoms was not related to the exposure level (e.g., EMF exposure vs. sham)

	Sample (included in analysis)	Exposure type	Experimental conditions, exposure duration and blinding status	Outcome measure	Result
VDU					
Andersson et al. [59]	17 IEI-EMF	VDU, MF: 245 nT (5 Hz – 2 kHz) and 19 nT (2 kHz – 400 kHz); EF: 7 V/m (5 Hz – 2 kHz) and 10 V/m (2 kHz – 400 kHz)	Before and after cognitive behavioural treatment: 1 exposure session and 1 sham session, each 30 min; sessions were separated by at least 1 week; double-blind study	Global rating of symptoms: assigned score on a 100-mm visual analogue scale (VAS)	No statistically significant effect of exposure
Flodin et al. [60]	15 IEI-EMF	Cathode ray tube (VDU or TV), MF: 5–2000 Hz mean of 342 nT, 2–400 kHz mean of 36 nT; EF: 5–2000 Hz mean of 288 V/m, 2–400 kHz mean of 6.2 V/m	2 exposure sessions to the same signal, 2 sham sessions, each up to 1 h; sessions were separated by 2–32 days; double-blind study	Chosen items from a questionnaire consisting of at least 29 symptoms	No statistically significant effect of exposure
Lonne-Rahm et al. [61]	24 IEI-EMF, 24 controls (12 IEI-EMF and 12 controls in each of two experiments that where combined for analysis)	VDU, MF: 198 nT (5 Hz – 2 kHz) and 18 nT (2 kHz – 400 kHz); EF: 12 V/m (5 Hz – 2 kHz) and 10 V/m (2 kHz – 400 kHz)	4 different exposure sessions: VDU “on” with stressor (visual test combined with calculations during a limited time period), VDU “off” with stressor, VDU “on” without stressor, VDU “off” without stressor, each 30 min; sessions were separated by 1 week; double-blind study	Assigned scores on a VAS for rating the severity of facial skin symptoms, stress level, tiredness	No statistically significant effect of exposure
Oftedal et al. [53]	20 IEI-EMF	VDU; static EF: − 2 – 9.5 kV/m, ELF EF: 2–12 V/m, VLF EF: 0.3–10.5 V/m	1 exposure session without filter, 1 exposure session with inactive filter, 1 exposure session with active filter, each session lasted 2 weeks; symptom registration each day in week 2 of each session; double-blind study	Assigned scores on a 10-interval scale for rating the severity of 7 categories of facial skin symptoms, 1 additional category for “other” symptoms	Symptoms less pronounced with active filters, small significant effect for sensations of tingling, itching, pricking (*p* = 0.03); no statistically significant effect for the 6 other groups of skin symptoms
Oftedal et al. [62]	38 IEI-EMF	VDU; ELF EF: 0–20 V/m, VLF EF: 0.05–1 V/m	1 exposure session without filter, 1 exposure session with inactive filter, 1 exposure session with active filter, each session lasted 3 months; symptom registration each day in week 4 and in the last week of each session; double-blind study	Assigned scores on a 10-interval scale for rating the severity of 13 symptoms (skin: 4 symptoms, eye: 5 symptoms, nervous system: 4 symptoms)	No statistically significant effect of exposure
Swanbeck and Bleeker [63]	30 IEI-EMF	VDU; static EF: VDU-A: 0.2 kV/m, VDU-B: 30 kV/m; MF (1–300 kHz): VDU-A: 50 nT, VDU-B: 800 nT, alternating EF: both VDUs about 60 V/m	1 exposure session with VDU-A, 1 exposure session with VDU-B, each 3 h; sessions were separated by 1 day; double-blind study	Report of any skin symptoms experienced during a trial	No effect of exposure, concluded without statistical analysis
ELF					
Kim et al. [64]	15 IEI-EMF, 16 controls	60 Hz MF, 12.5 μT at the subjects’ head	1 exposure session, 1 sham session, each 31 min; sessions were separated by at least 1 day; double-blind study	Assigned scores on a 4-point scale [40] for rating the severity of 8 symptoms (throbbing, itching, warmth, fatigue, headache, dizziness, nausea, palpitations)	No statistically significant effect of exposure
McCarty et al. [52]	1 IEI-EMF	60 Hz EF, average field of about 300 V/m around the head, less than 50 kV/m around the body	1 condition with alternating 100 ms “field on”-“field off”-pulses during 100 s, 1 condition with 100 s continuous exposure, 1 sham condition, test 1: “on-off” and the sham condition repeated 10 times; test 2: all conditions repeated 5 times; next trial delayed until subject reported that symptoms had abated; double-blind study	Verbal report of any symptoms experienced during a trial, questioned in interview	Statistically significant more severe symptoms reported in trials during pulsed exposure (*p* < 0.05), but not during continuous exposure
Szemersky et al. [65]	49 IEI-EMF, 57 controls	50 Hz MF, exposed arm: 500 μT, other body parts: 1.14 μT	10 exposure trials, 10 sham trials, each 1 min; trials were separated by 30 s; double-blind study	Assigned scores on a 4-point scale for rating the severity of 15 symptoms (nervous system: 4 symptoms; visceral functions: 3 symptoms; sensations in the exposed hand: 8 symptoms), 1 additional category for “other” symptoms	No statistically significant effect of exposure
Toomingas [66]	1 IEI-EMF	50 Hz MF, 34 or 100 μT	Exposure trials at 2 different intensities and for a duration of either 1 or 10 s, sham trials for either 1 or 10 s, each condition repeated 24 times; interval between trials not reported; single-blind study	Verbal report of any symptoms experienced during a trial	No effect of exposure, concluded without statistical analysis
Trimmel and Schweiger [55]	36 IEI-EMF, 30 controls	50 Hz MF, 1 mT in the head area	Each participant took part in 2 of the following sessions: session with EMF + noise, sham session (noise only), control session without noise and without EMF, each 1 h; sessions were separated by 1 h; double-blind study	Assigned levels in the “Befindlichkeitsskala” questionnaire [67] for rating mood	More discomfort during EMF + noise compared to noise alone condition across all participants (*p* < 0.05). No statistically significant effect for each group analyzed separately
Wenzel et al. [68]	3 IEI-EMF, 7 controls	50 Hz MF, 96 mT	1st protocol: 25 min “field on”, 25 min “field off”; 2nd protocol: every 5 min “field on” and “field off” for a total duration of 50 min; no interval between conditions; double-blind study	Verbal report of any symptoms experienced during a trial	No effect of exposure, concluded without statistical analysis
RF					
Augner et al. [56]	8 IEI-EMF, 49 controls	Environmental, mainly 900 MHz GSM downlink signal, low: 5.2 μW/m^2^, medium: 153.6 μW/m^2^, high: 2126.8 μW/m^2^	Each participant allocated to 1 of 3 exposure scenarios with different combinations of five 50-min sessions with low, medium or high exposure level; interval of 5 min between each session; double-blind study	Assigned scores on a 5-point scale for rating the severity of 3 items of well-being (good mood, alertness, calmness) from The Multi-Dimensional Well-Being questionnaire (MDBF) [69]	Main effects of exposure (all participants): Less psychological arousal (i.e., significantly calmer) with scenarios including high and medium exposure levels (*p* = 0.042). No statistically significant effects for other factors of well-being (good mood and alertness). No statistically significant interaction between exposure and group of participants (IEI-EMF or controls)
Barth [70]	1 IEI-EMF	Mobile phone (no information about exposure level, only that the mobile phone was switched “on” or “off”)	15 exposure trials, 16 sham trials; exposure duration and interval between trials not indicated; double-blind study	Verbal report of experiencing any of 4 symptoms during a trial: palpitations, chest pain, vertigo, prickling in the arm	No statistically significant effect of exposure
Eltiti et al. [21]	44 IEI-EMF, 114 controls	GSM 900 MHz, GSM 1800 MHz, UMTS base station signals; combined power flux density of 10 mW/m^2^ for GSM signal, 10 mW/m^2^ for UMTS signal	2 exposure sessions to GSM (combined signal of GSM 900 and GSM 1800 frequencies) or UMTS signal and 1 sham session, each 50 min; sessions were separated by at least 1 week; double-blind study	Assigned scores on a 100-mm VAS for rating the severity of 6 symptoms (anxiety, tension, arousal, relaxation, discomfort, and fatigue); assigned levels on symptom scales consisting of a list of 57 symptoms extracted from the Electromagnetic Hypersensitivity Questionnaire [6]	For individuals with IEI-EMF: elevated levels of arousal when exposed to a UMTS signal (*p* < 0.0025), likely due to a non-balanced design (45% of participants had UMTS exposure in the first of the three sessions): no statistically significant effect when conditions were compared for each session separately; no statistically significant effect for the 6 other items of subjective well-being, nor for symptoms
Furubayashi et al. [36]	11 IEI-EMF, 43 controls	2.14 GHz WCDMA base station signal with power density of 0.265 W/m^2^; EF at the subjects’ head: 10 V/m, calculated brain SAR_10g peak_: 0.0078 W/kg	1 session with continuous exposure, 1 session with intermittent exposure with EMF turned “on” and “off” randomly every 5 min (50% of the time "on"), 1 sham session, 1 noise session, each 30 min; two sessions on 1 day; sessions were separated by at least 2 h; double-blind study	Assigned level of discomfort on a 5-point scale; assigned scores on a 5-point scale in the Profile of Mood States (POMS) questionnaire [71] for rating the severity of 6 states of mood (tension-anxiety, anger-hostility, depression, vigor, fatigue, confusion)	No statistically significant effect of exposure
Hietanen et al. [57]	20 IEI-EMF	Analogue 900 MHz NMT phone (output power: 1 W), digital GSM phone 900 MHz (output power: 0.25 W), digital GSM phone 1800 MHz (output power: 0.125 W); power densities: 2–200 W/m^2^	3 exposure sessions to different signals, 1 sham session, each 30 min; sessions were separated by at least 60 min; double-blind study	Verbal report of any symptoms and sensations experienced during a trial	Higher number of symptoms was reported for the sham condition than for any of the RF exposures. Statistical significance (*p* < 0.05) of this effect was explicitly specified only for men.
Hillert et al. [39]	38 IEI-EMF, 33 controls	884 MHz GSM mobile phone-like signal, head (calculated): SAR_10g averaged peak spatial_ 1.4 W/kg	1 exposure session, 1 sham session, each 3 h; sessions were separated by at least 1 week; double-blind study	Assigned scores on a 7-point Likert scale for rating the severity of 14 symptoms (headache, fatigue, nausea, vertigo, difficulties concentrating, feeling low-spirited, temporary vision problems, 5 questions on dermal complaints, stress, heat or pain from the left ear), 1 additional category for “other” symptoms	Headache more commonly reported after RF exposure than after sham (*p* < 0.001) due to a difference between headache reports during RF and sham in the non-symptom group; no statistically significant effect on increase in percentage reporting headache. No effect of exposure for IEI-EMF participants and no statistically significant effect of exposure for other symptoms.
Kwon et al. [72]	17 IEI-EMF, 20 controls	1950 MHz WCDMA mobile phone-like signals (output power: 24 dB), head: SAR_1g_ 1.57 W/kg (measured and calculated)	1 exposure session, 1 sham session, each 31 min; sessions were separated by at least 1 day; double-blind study	Assigned scores on a 4-point scale [40] for rating the severity of 8 symptoms (throbbing, itching, warmth, fatigue, headache, dizziness, nausea, palpitation)	No statistically significant effect of exposure
Nam et al. [73]	18 IEI-EMF, 19 controls	835 MHz CDMA mobile phone (transmission power: 300 mW), SAR_1g_ 1.2 W/kg (according to manufacturer’s information)	1 exposure session, 1 sham session, each 31 min; sessions were separated by at least 1 day; single-blind study	Assigned scores on a 4-point scale [40] for rating the severity of 9 symptoms (redness, itching, warmth, fatigue, headaches, dizziness, nausea, palpitation, indigestion)	No statistically significant effect of exposure
Nieto-Hernandez et al. [58]	60 IEI-EMF, 60 controls	385.25 MHz continuous wave signal, 385.25 MHz TETRA handset-like signal (pulsing frequency of 16 Hz), output power each 250 mW; close to the antenna SAR_10g_ 1.3 W/kg	2 exposure sessions to different signals, 1 sham session, each 50 min; sessions were separated by at least 1 day; double-blind study	Assigned scores in the Positive and Negative Affect Schedule (PANAS) questionnaire [74]; assigned scores on an 11-point numerical scale for rating the severity of 8 symptoms (headache; fatigue; dizziness; nausea; sensations of warmth or burning on skin; skin itching, tingling, stinging or numbness; feeling irritable, anxious or depressed; difficulty concentrating or thinking)	*Before correction for multiple comparisons* For TETRA signal: increased difficulty concentrating. For continuous wave signal: increased ratings of headache in all participants (*p* = 0.004), fatigue showed reduced initial rating (*p* < 0.0001) and faster increase (*p* = 0.014) in non-sensitive participants, increased difficulty concentrating (*p* = 0.037) and reduced sensations of itching in IEI-EMF individuals. *After correction for multiple comparisons* No statistically significant effect of exposure for TETRA signal. For continuous wave signal: reduced sensations of itching in IEI-EMF individuals (*p* = 0.03)
Oftedal et al. [22]	17 IEI-EMF	902.4 MHz GSM mobile phone-like signal, maximum output power 23 dBm ( ~̴0.2 W), head SAR_10g peak spatial_ = 0.8 W/kg	Up to 4 exposure sessions and up to 4 sham sessions, each 30 min, sessions were separated by at least 2 days; double-blind study	Assigned scores on a 100- mm VAS for rating the severity of pain/discomfort in the head and “other” symptoms	No statistically significant effect of exposure
Regel et al. [23]	33 IEI-EMF, 84 controls	2.140 MHz UMTS base station-like signal, 1 V/m (brain SAR_10g peak spatial_: 45 μW/kg (calculated)) or 10 V/m (brain SAR_10g peak spatial_: 4500 W/kg (calculated))	2 exposure sessions to different intensities, 1 sham session, each 45 min; sessions were separated by 1 week; double-blind study	Scores in the questionnaire on the Current Disposition [75]; scores in the modified Quality-of-Life questionnaire [76] (23 items within 5 subscales: anxiety, somatic symptoms, inadequacy, depression, hostility)	No statistically significant effect of exposure
Rubin et al. [77]	60 IEI-EMF, 60 controls	900 MHz GSM mobile phone, continuous wave signal, SAR 1.4 W/kg (near to the antenna)	2 exposure sessions to different signals, 1 sham session; each 50 min; sessions were separated by at least 1 day; double-blind study	Assigned scores on a 100-mm VAS for rating the severity of 7 symptoms (headaches; nausea; fatigue; dizziness; skin itching, tingling, or stinging; sensations of warmth or burning on skin; eye pain or dryness)	No statistically significant effect of exposure
Verrender et al. [78]	3 IEI-EMF	902–928 MHz RF signal, average output power: 1 W; power density: 0.3 W/m^2^	6 exposure sessions and 6 sham sessions, each 30 min; separated by at least 1 h; double-blind study	Assigned scores on a 100-mm VAS for rating the severity of the most immediate symptom triggered during the open provocation	No statistically significant effect of exposure
Wallace et al. [79]	48 IEI-EMF, 132 controls	420 MHz TETRA base station signal, 10 mW/m^2^, estimated SAR: 271 μW/kg	1 exposure session, 1 sham session, each 50 min; sessions were separated by at least 1 week; double-blind study	Assigned scores on a 100- mm VAS for rating the severity of 6 symptoms (anxiety, tension, arousal, relaxation, discomfort, and fatigue); assigned levels on symptom scales consisting of a list of 57 symptoms extracted from the Electromagnetic Hypersensitivity Questionnaire [6]	No statistically significant effect of exposure
Wilén et al. [33]	20 IEI-EMF, 20 controls	900 MHz GSM mobile phone-like signal, SAR_10g_: 0.8 W/kg (calculated)	1 exposure session, 1 sham session, each 30 min; sessions were separated by at least 1 day; single-blind study	Report of any symptoms experienced during a trial using a follow-up form	No statistically significant effect of exposure
Various					
Rea et al. [54]	Experiment 1: 100 IEI-EMF; Experiment 2: 25 IEI-EMF, 25 controls; Experiment 3: 16 IEI-EMF	MF pulses of various frequencies between 0.1 Hz and 5 MHz, at hand level: 70 nT, at knee level: 350 nT, at floor level: 2900 nT	Experiment 1 and 2: 21 exposure trials at different frequencies, 5 sham trials; experiment 3: 1 exposure trial and 5 sham trials on two separate occasions, each 3 min; interval between trials not reported	Report of any symptoms experienced during a trial	16 out of 100 participants reported consistent reactions to exposure in all three experiments, but not to sham; no statistical analysis

### Methodological quality

#### Rating of risk of bias and imprecision

**Fig. 2:**
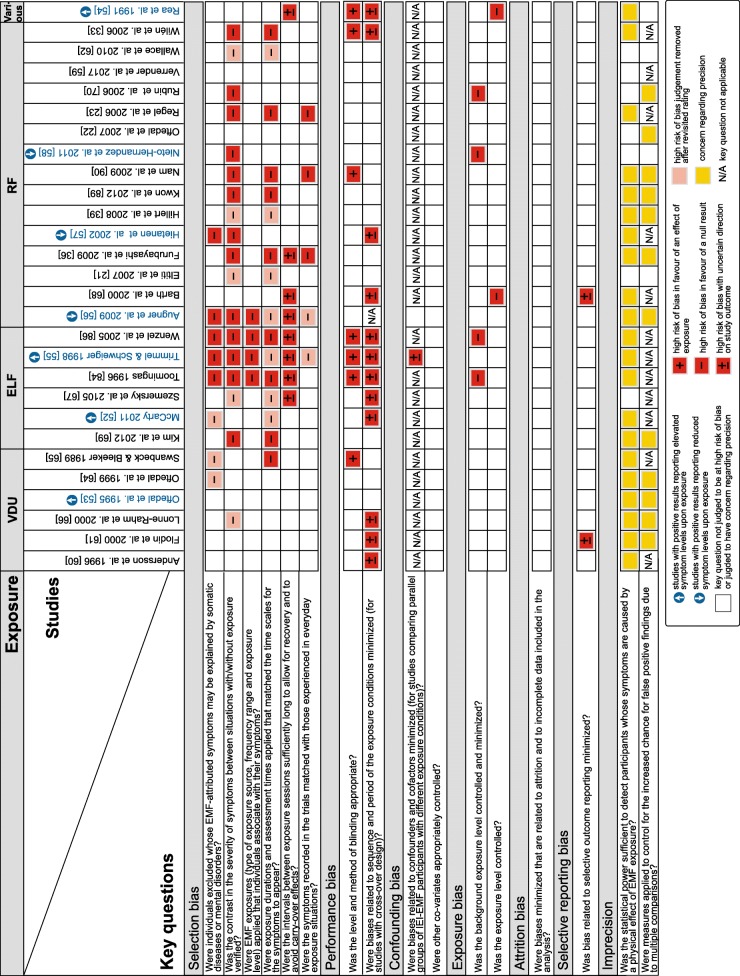
Key questions judged to be at high risk of bias or judged to have concern regarding precision. The ratings are depicted for individual studies. Studies in blue: indicated an effect of exposure; studies in black: indicated no effect of exposure. Augner et al. [56] and Trimmel and Schweiger [55] reported effects of exposure for the combined group of individuals with IEI-EMF and healthy controls. The remaining studies with positive results reported effects of exposure for IEI-EMF individuals only

Additional file 1: Table S2 and Additional file 2 depict the results for the rating of 16 key questions, based on the extracted data of the 28 included studies. Key questions judged to be at high risk of bias or judged to have concern regarding precision in data analysis are depicted in Fig. 2 for individual studies.

The most common methodological limitations were related to the selection of participants, performance and imprecision: 23 (82%) studies were susceptible to selection bias (i.e., at least one key question within this domain was judged to be at high risk of bias), 14 (50%) studies to performance bias and 23 (82%) studies were judged to have concern regarding precision (Fig. 2).

Under *selection bias*, three key questions were frequently judged to be at high risk of bias. Eight (29%) studies did not consider a pre-screening to exclude individuals whose EMF-attributed symptoms may be explained by somatic diseases or mental disorders. Further, 18 (64%) studies did not verify the contrast in the severity of symptoms between situations with and without exposure as a basis for the selection of participants. Also, 16 (57%) studies used pre-defined exposure durations and assessment times and did not verify the match with individual time scales for the symptoms to appear.

Under *performance bias,* high risk of bias was identified in seven (25%) studies, in which the level and method of blinding may not have been effective. Twelve (43%) studies were further susceptible to period or sequence effects.

The key questions addressed under *confounding bias*, *exposure bias, attrition bias,* and *selective reporting bias* were less frequently judged to be at high risk of bias.

The total number of key questions judged to be at high risk of bias varied between zero and nine across the individual studies. In three (11%) studies, none of the key questions related to risk of bias were judged to be at high risk [22, 53, 78], while in 15 (54%) studies, three or more sources of high risk of bias were identified (Fig. 2).

*Imprecision* in data analysis was identified in many of the reviewed studies because they did not e.g. provide a statistical power estimate (*n* = 21, 75%) and/or adjust for multiple comparisons when this was relevant (*n* = 12, 43%). The total number of key questions judged to have concern regarding precision varied between zero and two across the individual studies. Five (18%) studies were not judged to have concern regarding precision, while 13 (46%) studies were judged to have concern regarding one key question and 10 (36%) studies regarding both key questions (Fig. 2).

#### Comparison of the profiles of limitations between studies with positive and negative results

**Fig. 3:**
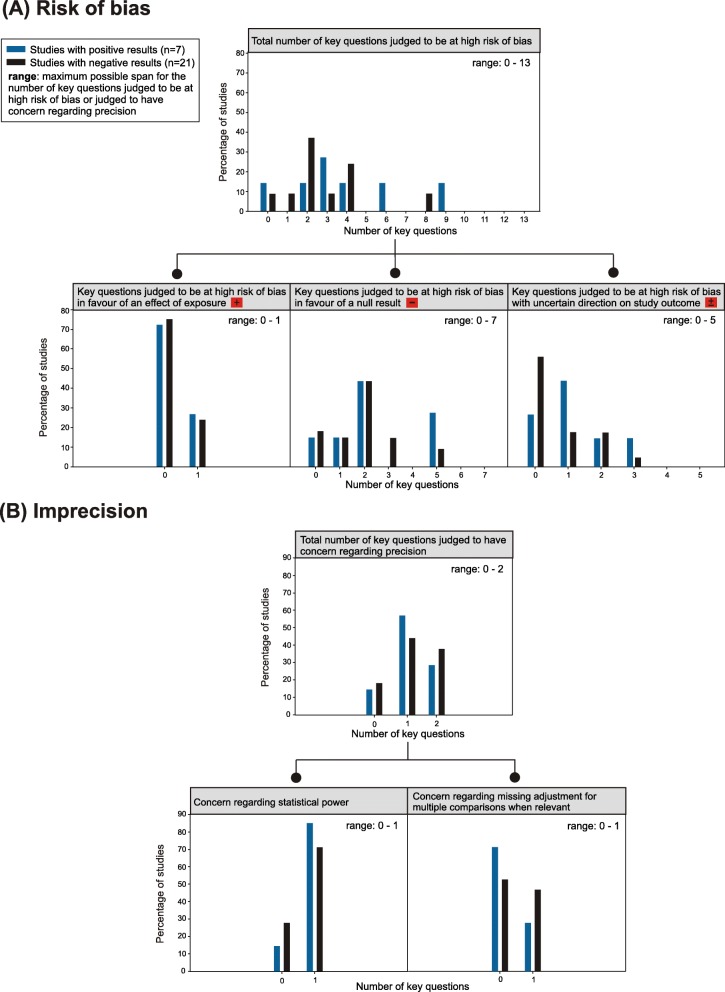
Comparison of the profiles of limitations between studies with positive and negative results. The distributions are depicted in percentages and are sorted by study outcome. **a** Distributions for judgements about risk of bias. Upper figure: total number of key questions; lower figures: numbers of key questions according to direction of bias. See Table 1 for specification which key questions are grouped under the various directions of bias. **b** Distributions for judgements about imprecision. Upper figure: total number of key questions; lower figures: left – concern regarding statistical power, right – concern regarding missing adjustment for multiple comparisons

The statistical comparisons between studies with positive and negative results regarding the distributions of the key questions judged to be at high risk of bias or judged to have concern regarding precision yielded small to moderate differences. Also, the variability within each group was large (Fig. 3 and Table 3).

**Table 3:** Statistical comparison of the profiles of limitations between studies with positive and negative results

	Risk of bias				Imprecision	
	Total number of key questions judged to be at high risk of bias	Number of key questions judged to be at high risk of bias in favour of a null result	Number of key questions judged to be at high risk of bias with uncertain direction on study outcome	Studies with key question judged to be at high risk of bias in favour of an effect of exposure	Studies judged to have concern regarding statistical power	Studies judged to have concern regarding missing adjustment for multiple comparisons when relevant
	Median (Interquartile range)	Median (Interquartile range)	Median (Interquartile range)	n (%)	n (%)	n (%)
Studies with positive results	3.0 (4.0)	2.0 (4.0)	1.0 (2.0)	2 (29%)	6 (86%)	2 (29%)
Studies with negative results	2.0 (2.0)	2.0 (1.5)	0 (1.5)	5 (26%)	15 (71%)	10 (48%)
Test statistics and p-values	Mann-Whitney-U: U = 55.5 *p* = 0.35^1,4^	Mann-Whitney-U: U = 65.0 *p* = 0.68^2,4^	Mann-Whitney-U: U = 54.5 *p* = 0.32^2,4^	Fisher’s exact: *p* = 1.00^2^	Fisher’s exact: *p* = 0.64^3^	Fisher’s exact: *p* = 0.66^3^

Significance levels: ^1^α = 0.05, ^2^α = 0.017, ^3^α = 0.025

^4^Exact significance

Studies with negative results had poorer rating than studies with positive results only for the key question related to concern regarding “missing adjustment for multiple comparisons when relevant”. The *p*-values for all performed statistical comparisons were between 0.32 and 1.00. Similar results were found when removing from the analysis the three studies that suggested a protective effect of exposure [56–58].

## Discussion

The goal of this systematic literature analysis was to evaluate methodological limitations that might have fostered false positive or false negative results in experimental provocation and intervention studies which tested the effect of VDU, ELF, or RF-EMF exposure on symptom development in individuals with IEI-EMF. Using a customized rating tool, we identified sources of risk of bias and imprecision in individual studies. Further, we compared the credibility between studies suggesting an effect of exposure and studies indicating no effect of exposure.

### Summary of evidence

Seven (25%) studies included in this review reported elevated or reduced symptom levels upon exposure to EMF, while the majority of the studies (*n* = 21, 75%) did not find evidence for exposure-related effects in IEI-EMF individuals. Study outcomes, i.e., positive or negative results, were not restricted to specific types or frequency ranges of exposure.

#### Risk of bias and imprecision

Most common across the reviewed studies were limitations regarding the selection of study participants or the matching of study design to the participants, the counterbalancing of the exposure sequence and the effectiveness of blinding. Further, many studies possibly suffered from low statistical power. Therefore, we will discuss these key questions in more detail.

In 23 (82%) studies, the *selection* of study participants might have introduced substantial bias in the results. These studies did not carefully assess potential participants before enrolment in the study (i.e., at least one key question within the domain *selection bias* was judged to be at high risk of bias). Heterogeneous study groups might have been the result of such selection procedures and the applied exposure parameters were probably not appropriate for all participants, which might in some cases have been reasons for false negative results. However, five (18%) studies had applied strict inclusion criteria in an effort to identify individuals with symptoms that could be related to EMF exposure and ensured that the participants and the design of the experiments were matched [22, 53, 59, 60, 78]. It is important to note that four [22, 59, 60, 78] of these studies were unable to find an effect of exposure on well-being or symptom development.

One specific limitation in the selection procedure relates to a lack of screening for somatic diseases or mental disorders that may explain the EMF-attributed symptoms. Eight (29%) of the reviewed studies did not apply criteria to identify and exclude individuals whose health complaints are likely unrelated to EMF exposure. Baliatsas et al. [37] noted that the inclusion of individuals whose symptoms are not related to EMF exposure may dilute the findings and reduce the chance of identifying individuals who suffer from health complaints due to a physical effect of EMF exposure. It is, however, challenging to diagnose whether somatic diseases and mental disorders are definitive medical explanations of the reported symptoms. Also, hypersensitivity to EMF might be comorbid in some individuals. Nevertheless, to avoid an underestimation of a potential effect of exposure, a rigorous anamnesis is required to identify and exclude individuals who misattribute their symptoms to EMF-exposure. Likewise, one can argue that the unintentional inclusion of healthy individuals in the group of IEI-EMF individuals may dilute the results and favour non-significant statistical results. Interestingly, however, the two studies that concluded about an effect of exposure only based on the analysis of the combined groups of individuals with IEI-EMF and healthy controls [55, 56] are not consistent with this hypothesis. The authors reported statistically significant results, i.e., protecting or hazardous effects of exposure, for the combined groups. While Augner et al. [56] did not conduct a separate analysis for each group, Trimmel & Schweiger [55] could not demonstrate an effect of exposure when the two groups were analysed separately. Furthermore, Hillert et al. [39] reported an effect of exposure only for healthy participants and not for the group of individuals who reported suffering from hypersensitivity to EMF. There is no straightforward explanation as to why in some studies healthy participants appear to react more to EMF exposure than those participants who attribute their symptoms to EMF. Also, a protective effect is difficult to explain. On the one hand, both bias and chance might have played a role, while on the other hand, we cannot rule out that, among the healthy participants, there might have been individuals reacting to EMF who did not know that they were sensitive. However, even if that was the case, it is unlikely that this alone could explain the results.

A further common weakness in the selection procedure relates to the verification of the contrast in the development of symptoms between situations with and without exposure, which was not considered in 64% (*n* = 18) of all studies. Consequently, the number and severity of the symptoms reported in the experimental sessions might partly or completely have been unrelated to the EMF exposures, which could have reduced the power to detect potential effects of exposure.

A limitation in the selection procedure was also identified with regard to exposure durations and assessment times. Fifty-seven percent (*n* = 16) of the studies did not consider individual latencies in symptom onset. Thus, it is unclear whether the application of standardized exposure durations and assessment times were sufficient to provoke symptoms in each participant. Time scales in symptom development may greatly differ among individuals with IEI-EMF. In a questionnaire study by Röösli et al. [3], respondents reported periods of a few minutes up to several days for the symptoms to appear.

Although 23 (82%) studies ensured that the symptoms recorded during the experimental session were matched with the symptoms experienced in everyday life, many different tools (e.g., validated or customized self-report questionnaires) were used to record the symptoms. However, validated questionnaires developed for studies with IEI-EMF individuals [6], have been rarely used [21, 79]. As the lack of standardization makes it difficult to compare studies, validated tools are preferable but priority should be given to the match of the symptoms recorded in the experimental session with those experienced in everyday exposure situations.

*Performance bias* also lowered the credibility of the results in some studies. One of these limitations relates to the level and method of blinding. Twenty-six (93%) of the studies stated that both participants and the research personal were blinded to the exposure status (double-blind), which is commonly considered low risk of bias [47]. However, we regarded the blinding to not be adequately ensured in seven (25%) of all reviewed studies because clues that might reveal the exposure status were not sufficiently controlled and it was not reported that tests were done to control whether the blinding was effective. Four studies demonstrated that belief or knowledge about the exposure status may play a significant role in the development of symptoms in IEI-EMF individuals (see below). Thus, any audible, visible or tactile clues might disclose the exposure status and thereby provoke more severe or a higher number of symptoms during EMF exposures than during sham exposures.

Furthermore, possible period and sequence effects might have either masked real effects of exposure or fostered false effects. Sequence effects were likely prevented in most studies by applying sufficiently long intervals between the different exposure conditions. In 12 (43%) of the studies, however, there were significant deviations from counterbalancing of the exposure conditions without controlling for potential period effects in the statistical analysis. In the study by Hietanen et al. [57], it is likely that a period effect might have been the reason for the unexpected finding of a higher number of symptoms during sham exposure than during EMF exposure. The sham exposures were always presented first or second within a series of four trials, and physiological testing also suggested higher stress levels in the initial phase of the experiment.

Our evaluation further yielded that 21 (75%) of the reviewed studies possibly suffered from low statistical power. These studies did not provide statistical power estimates, although some of them included a relatively high number of participants. Nevertheless, also for the studies with a high number of participants, it remains unclear whether the statistical power was high enough to detect a potential effect of exposure. For the studies with few participants and a low number of trial repetitions, the statistical power was probably far too low and the risk for false negative results high. Interestingly, however, Eltiti et al. [80] performed an aggregated analysis to increase the statistical power by combining data from two studies [21, 79], but could not reveal any statistically significant effect of exposure for double-blind experimental sessions. The aggregated analysis included 88 IEI-EMF participants and the statistical power was estimated to be 0.82 to detect a small effect and 0.99 to detect a medium effect.

Looking at the risk of bias and imprecision assessment for individual studies, three (11%) of the reviewed studies [22, 53, 78] were judged to be free from sources of risk of bias, although the study by Oftedal et al. [53] was judged to have concern regarding precision because the authors did not provide a statistical power estimate and did not adjust for multiple comparisons. However, since only one of seven groups of symptoms reached statistical significance (*p* = 0.03), but close to the significance threshold, the results would not have been regarded as statistically significant if adjustment for multiple comparisons had been applied. Verrender et al. [78] analyzed individual data obtained from three participants, each tested under a series of trials consisting of a sufficient number of repetitions for the exposure and sham condition to ensure a statistical power of 0.80. Although their approach was suited to detect potential effects of exposure, generalization of the results to other individuals with IEI-EMF is not possible with such a low number of participants. In about half of the reviewed studies (15 out of 28) we identified three or more methodological limitations. These limitations lowered the credibility of their results, i.e., they might have given rise to either false positive or false negative results.

#### Comparison of the profiles of limitations between studies with positive and negative results

The distributions of key questions judged to be at high risk of bias or judged to have concern regarding precision were almost comparable between studies with positive and negative results. Furthermore, there was no statistically significant association between the direction of bias and study outcomes. This was not expected because it would be more plausible that mainly key questions judged to be at high risk of bias in favour of a null result would be identified in studies suggesting no effect of exposure and that mainly key questions judged to be at high risk of bias in favour of an effect of exposure would be identified in studies with positive results.

For imprecision, the results were also contrary to what was expected. Studies reporting an effect of exposure were more often judged to have concern regarding statistical power, although a low statistical power decreases the likelihood of detecting an effect of exposure, while studies reporting no effect of exposure were more often judged to have concern regarding missing adjustment for multiple comparisons, although missing adjustment increases the likelihood of detecting an effect of exposure.

Although the analyses at group level could not provide evidence that particular limitations, i.e., risk of bias or imprecision in data analysis, explain why some studies suggest an effect of exposure while others did not, the large variability in the distributions suggests that bias and imprecision might have affected the outcomes of at least some studies in both groups.

A limitation concerning the performed statistical analyses was the very low statistical power. The low power was to a large extent due to the restricted number of studies eligible for this review, especially those with a positive outcome, and the large variability in the distribution of key questions judged to be at high risk of bias (Fig. 3 and Table 3). Low statistical power would also be expected for the analyses based on Fisher’s exact test. This means that the likelihood would be low to detect statistically significant differences between the two study groups - even if the differences in the distributions of key questions (e.g., regarding the direction of bias) were large enough to be considered as crucial factors for study outcomes. Therefore, we should not solely base our conclusions on the results of statistical significance tests, but also consider the effect sizes; and for the analysis where a particular direction of bias on the study outcome was expected, look at the direction of the effect.

Another possible reason for the lack of statistical significance between the two study groups might be that some biases probably had a larger effect on the outcomes than others. Also, because it is about *risk* of bias, when a key question is judged to be at high risk of bias, e.g., due to concerns regarding blinding, a bias may occur in some studies, but not necessarily in others, and the number of participants affected by it may vary and therefore also the impact the bias has on the study outcome. Furthermore, when revisiting the assessment tool and the results of the assessment, we noticed that the use of an appropriate method to address a particular key question or additional results or information provided by the studies might in some cases have an influence on the rating of other key questions. Therefore, in a revisited rating, we assessed such interferences that were not considered in the rating tool, but allowed removal of high risk of bias judgements for some key questions (Fig. 2). We identified one case where the use of an appropriate method to address a key question influenced the rating of another key question: if a sufficient contrast in symptom development between situations with and without exposure is confirmed for everyday exposure situations, it is less likely that the lack of criteria for the exclusion of individuals whose symptoms may be explained by somatic diseases or mental disorders would result in a high risk of bias, although indicated as such in the initial rating. This was the case for three studies [52, 62, 63]. Furthermore, we identified three cases where additional results or information provided by the studies have implications for the risk-of-bias ratings of other key questions. First, three studies [21, 61, 79] included open provocation tests that were analyzed at a group level and in which the participants were informed about when they were exposed and when not. In this situation, IEI-EMF individuals reported significantly more severe symptoms during exposure than during sham as a group, but such differences between exposure conditions were not observed in the double-blind trials. The open provocation tests were not part of the selection procedures. However, at group level, the results suggest that the contrast in symptom severity was high enough to reveal changes between different exposure conditions and that the exposure durations/assessment periods were long enough for the symptoms to appear/to be detected. Thus, in studies including open provocation tests that were analyzed at a group level, high risk of bias judgements could be removed for these two key questions. Second, this also applies to the two studies that demonstrated a significant correlation between the number or severity of the symptoms and the IEI-EMF individuals’ belief of being exposed irrespective of the actual exposure status [39, 65]. Third, three of the studies reporting an effect of exposure [52, 55, 56] did not provide information indicating that the symptoms recorded in the experimental sessions are relevant for IEI-EMF individuals (i.e., matched with those experienced in everyday life) or that the exposure durations and assessment periods were long enough. Still, the reported relation between EMF exposure and severity of symptoms suggests that the standardized experimental conditions were appropriate for the included participants at a group level such that high risk of bias judgements could also be removed for these two key questions.

All adjustments made during the revisited rating concerned selection biases in favour of a null-result (Fig. 2). After these adjustments, six studies, three with positive results [52, 55, 56] and three with negative results [61, 63, 65], had a lower number of key questions judged to be at high risk of bias compared to the initial rating and four additional studies [21, 39, 62, 79], all with a negative result, had no remaining key questions judged to be at high risk of bias. However, the revisited rating resulted only in minor changes in the distributions of key questions judged to be at high risk of bias and also the statistical comparisons between studies with positive and negative results yielded similar results to the comparisons before these adjustments.

#### Physical effects of exposure vs. the nocebo effect

From the reviewed studies there is at present no reliable evidence for an effect of exposure. Nine of the included studies in this review suggested that a nocebo effect may explain the development of symptoms [22, 39, 59, 64, 65, 70, 77–79]. The symptoms correlated with beliefs and knowledge about being exposed, and this has been easy to demonstrate in experimental studies while it proved difficult to find reliable evidence for a physical relation between EMF exposure and health complaints. Note that the nocebo effect does not per se exclude the existence of a potential physical effect. However, the results obtained with previous research designs indicate that if a physical effect of exposure exists, it seems to be much weaker than the nocebo effect. Thus, the nocebo effect could either overshadow physical effects, add to symptoms provoked by somatic diseases or mental disorders or may otherwise be the only reason why symptoms are experienced in everyday life.

#### Recent development in IEI-EMF research

It is noteworthy that the interest in IEI-EMF research appears to have faded and comparatively few studies have been published in the past 5 years. Also, while the condition was reported by a relatively high proportion of the population (1.5–13.3%) in early surveys [81–85], a population-based survey in Taiwan found that the percentage of those who report suffering from IEI-EMF significantly decreased within a period of 4 years (from 13.3% in 2007 to 4.7% in 2011) [86]. The authors further noted a decline in the prevalence rate of the condition from 2007 to 2013 in the international literature. It is possible that the decline is partly due to official statements issued by the WHO [4] and SCENIHR [13] as well as the decreased amount of research activity in this area, resulting in less public concern for this topic. This would support the notion of a role of media reports for the development of IEI-EMF [25, 27].

### Strengths and limitations of this review

A number of strengths and limitations need to be addressed when interpreting the results of this review. The conclusions of this systematic review are based on the studies which were selected by using the outlined search strategy and inclusion criteria. Because we only considered peer-reviewed articles written in English and German in our analysis, it is possible that we might have missed some articles published in other languages and articles which did not undergo a peer-review process (grey literature). It is also possible that relevant search terms could not be found in the title, abstract or MeSH terms such that the searches in major literature databases did not identify all potentially relevant articles. However, given the large number of reviewed studies, it is unlikely that the inclusion of further experimental studies – peer-reviewed or grey literature – would alter our conclusions and the identified research needs.

Our primary inclusion criterion (“studies examined the well-being or the number/severity of symptoms upon exposure to EMF”) ruled out the evaluation of objective measures of health effects, i.e., studies investigating physiological or cognitive parameters, including blood pressure, heart rate, electrical activity of the brain and visual attention. Furthermore, our assessment was restricted to experimental studies investigating acute and semi-acute effects of exposure but did not consider observational epidemiological studies. The conclusions of this systematic review may thus not apply to objective outcomes, nor do they have implications for potential chronic effects of exposure to EMF. For review of observational studies on symptoms attributed to EMF see Baliatsas and Rubin [87] and Baliatsas et al. [88].

A strength of this review is that it evaluated a large number of studies and systematically assessed the methodological quality of individual studies using a rating tool customized to experimental studies on symptom development in individuals with IEI-EMF. A strong emphasis of the rating tool was on potential selection bias of the included participants because this is, in our opinion, a crucial aspect in IEI-EMF research. We assessed more key questions within this domain than other risk-of-bias rating tools (e.g., [47, 89]) and the emphasis on selection bias might have affected the conclusions of this evaluation. Furthermore, we initially assessed the 16 key questions related to risk of bias and imprecision independent of each other, but did a revisited rating in which we also evaluated interferences between these key questions and considered additional results in the studies that provided information about reduced risk of bias for some key questions (see Comparison of the profiles of limitations between studies with positive and negative results). While this probably resulted in a more correct picture of the methodological quality of individual studies, this was not done as systematically as the initial rating, i.e., these criteria were not specified prior to data extraction. Still, the same assessment standards were applied to all studies. Our analyses are further limited by the fact that we focused on methods that can be considered a source of high risk of bias, but we did not separate between low and moderate risk of bias, which would have provided a more rigorous assessment of the methodological quality of individual studies.

### Research needs

At present, it is not clear whether further provocation and intervention studies would lead to new insight that may provide a basis for definite conclusions about whether factors unrelated to EMF (e.g., the nocebo effect, somatic diseases or mental disorders) are responsible for the development of symptoms or whether there also is a physical relation between EMF exposure and health complaints in some individuals. Because there is no objective case definition for IEI-EMF (i.e., we lack diagnostic criteria and precise standards) and because potential effects may be weak, it is particularly challenging to recruit and select study participants in order to determine if any of the individuals with IEI-EMF might actually suffer from health complaints due to a physical effect of EMF exposure and even concentrated efforts to do so may not necessarily prove successful.

Dieudonné [90] recently noted that little progress has been made with an objective case definition for IEI-EMF. The key underlying problem is that its definition is seemingly circular: a precise definition of what is considered IEI-EMF is required to conduct rigorous experimental studies, but rigorous experimental studies are required to verify whether such a precise definition actually exists.

In consideration of the fact that we identified a limited number of methodically sound studies, and even though their results mainly indicate no effect of exposure, further attempts could be made to add high-quality studies to identify hypersensitive individuals, if they exist. This could be achieved by performing experiments at the individual level, but a large sample size and many repetitions of the same experimental condition would be needed to ensure statistical power and external validity. If it was possible with such studies to identify individuals who show a clear contrast in symptom development between situations with and without exposure in the double-blind trials, this could be an important step to provide evidence for a physical relation between EMF exposure and health complaints. Still, the potential for new insight provided by any further study and the clinical significance of its outcome should be weighted against time investment and required resources.

In planning any new experimental studies with IEI-EMF individuals, researchers have to be aware that, at this time, no ideal study design – if one exists – can be proposed. However, we recommend that any new studies aim to achieve a high credibility of the results by minimizing sources of risk of bias and imprecision. Properly addressing the 16 key questions outlined in our rating tool will contribute to reducing the likelihood for false positive or false negative results. Because participants will be aware of the fact that they may be exposed, it is not possible to eliminate the nocebo effect. Stress or anxiety in the experimental situation may equally provoke symptoms and reduce well-being, the consequence of which might be the masking of any potential effect of exposure. Future studies should therefore try to minimize the stress level through e.g. habituation sessions or other approaches such as at-home testing [91].

Because of the various factors that may provoke the symptoms, the group of individuals who attribute them to EMF exposure appears to be heterogeneous [29, 37, 42]. Therefore, also systematic reviews of studies that characterized groups of individuals with IEI-EMF may be useful to define subgroups and to form a better basis for effective treatment concepts. Some individuals with IEI-EMF also report suffering from health issues caused by other environmental exposures (e.g., chemicals, noise, odours) that fall under the definition of IEI [92, 93]. Thus, with regard to the identification of factors that provoke the symptoms as well as the development of treatment concepts, IEI-EMF should likewise be addressed in the context of other environmental intolerances.

Finally, any new review on studies that exposed or characterized groups of individuals with IEI-EMF should be prepared systematically and include an assessment of the methodological quality of the reviewed studies. A number of systematic reviews have been conducted for experimental studies with IEI-EMF individuals [14–19], but only two of these analyses [16, 19] assessed the methodological quality of individual studies. While synthesizing the results of several studies may strongly underpin the evidence for or against an effect, assessing the methodological quality additionally will help in judging the quality of this evidence.

## Conclusion

Seven of the 28 reviewed studies reported either a hazardous or a protective effect of EMF on individuals with IEI-EMF, while the majority of studies could not find evidence for an effect of exposure. Our analysis showed that both studies with positive and negative results suffered from methodological limitations that lowered credibility of the results. Limitations in design, conduct and analysis could therefore have given rise to either false positive or false negative results. Based on the assessment of the methodological quality of the reviewed studies in terms of risk of bias and imprecision, a limited number of studies – indicating that effects of exposure are unlikely – were judged to be methodically sound. Although some of these studies suggested sufficient statistical power, one cannot exclude the possibilities that either very weak physical effects of exposure or a few individuals that genuinely react to EMF remained undetected.

Given that the group of individuals suffering from IEI-EMF appears heterogeneous and given the evidence that the nocebo effect or medical/mental disorders may explain the health complaints in many individuals, future research should aim at exploring the various factors that may be important for developing IEI-EMF and for provoking the symptoms. This may form a basis for more efficient and individual treatment concepts. At present, it is not clear whether further provocation or intervention studies would provide new insight, but if further experimental studies are conducted, they should preferably be performed at the individual level. In order to increase the likelihood of detecting hypersensitive individuals, if they exist, we encourage researchers to achieve a high credibility of the results by minimizing of sources of risk of bias and imprecision. In any such study, efforts must be made to identify and include any individuals whose symptoms are caused by physical effects of the EMF exposure. A promising approach could also be conducting systematic reviews of studies that characterized groups of individuals with IEI-EMF in order to define subgroups and to explore this condition in the context of other idiopathic environmental intolerances.

## Additional file

Additional file 1: Supplementary material including the search strings and links for repeating the literature search in the electronic databases, a **Table S1.** with a rationale for rating the key questions related to risk of bias and imprecision and a **Table S2.** with the ratings of the methodological quality of individual studies. (PDF 296 kb)

Additional file 2: Aggregated ratings of 16 key questions. The key questions are grouped under six domains for risk of bias and one domain for imprecision. Included are the ratings for 28 studies. Some studies applied two or more methods to address a key question; they are therefore included more than once in the rating of a single key question. (PDF 252 kb)

## Data Availability

The data reported in this review are available from the publications and URLs cited.

## Abbreviations

CDMA: Code-Division Multiple Access
EF: Electric field
EHS: Electromagnetic hypersensitivity
ELF: Extremely low frequency field
EMF: Electromagnetic field
GHz: Gigahertz
GSM: Global System for Mobile Communications
Hz: Hertz
ICD: International classification of diseases
IEI-EMF: Idiopathic environmental intolerance attributed to electromagnetic fields
kHz: Kilohertz
MF: Magnetic field
MHz: Megahertz
mT: Militesla
NMT: Nordic mobile telephone
PICOS: Participants, intervention, comparison, outcome, and study design
PRISMA: Preferred reporting items for systematic reviews and meta-analyses
RF: Radiofrequency field
SCENIHR: European Scientific Committee on Emerging and Newly Identified Health Risks
TETRA: Terrestrial truncated radio
UMTS: Universal mobile telecommunications system
V/m: Volt/meter
VAS: Visual analogue scale
VDU: Visual Display Unit
VLF: Very low frequency field
WCDMA: Wideband code division multiple access
WHO: World Health Organization
μT: Microtesla
