# Supplementary material for: Methodological limitations in experimental studies on symptom development in individuals with idiopathic environmental intolerance attributed to electromagnetic fields (IEI-EMF) – a systematic review
Source: Environ Health. 2019 Oct 22;18:88. doi: 10.1186/s12940-019-0519-x (PMC6805477; doi:10.1186/s12940-019-0519-x)
Supplement: Supplementary file 1 — Supplementary material including the search strings and links for repeating the literature search in the electronic databases, a Table S1. with a rationale for rating the key questions related to risk of bias and imprecision and a Table S2. with the ratings of the methodological quality of individual studies. (PDF 296 kb) [file 12940_2019_519_MOESM1_ESM.pdf]

SUPPLEMENTARY MATERIAL

Methodological limitations in experimental studies on symptom development in individuals with idiopathic environmental intolerance attributed to electromagnetic fields (IEI-EMF) – A systematic review

Kristina Schmiedchen, Sarah Driessen, Gunnhild Oftedal

Literature search

The electronic database searches can be repeated using the following links and search strings:

PubMed
https://www.ncbi.nlm.nih.gov/pubmed/
(“electromagnetic hypersensitivity” OR electrohypersensitivity OR electrosensibility OR IEI-EMF OR “idiopathic environmental intolerance” OR “environmental intolerance” OR electrosensitivity OR electrosensitive OR “electric sensitivity” OR “Electrical sensitivity” OR “electromagnetic sensitivity” OR “electromagnetic field sensitivity” OR EHS) AND (well-being OR illness OR ill-health OR symptom* OR health OR health complaint* OR headache*) AND (exposure OR provocation OR mobile phone* OR cell phone* OR visual display unit* OR powerline* OR base station* OR GSM OR UMTS OR TETRA OR electromagnetic OR electric OR electrical OR magnetic OR radiofrequency)

WEB OF SCIENCE
http://apps.webofknowledge.com/WOS_AdvancedSearch_input.do?SID=1Dj2pmRuzWDH2Qn9I2y&product=WOS&search_mode=AdvancedSearch
TS = (“electromagnetic hypersensitivity” OR electrohypersensitivity OR electrosensibility OR IEI-EMF OR “idiopathic environmental intolerance” OR “environmental intolerance” OR electrosensitivity OR electrosensitive OR “electric sensitivity” OR “Electrical sensitivity” OR “electromagnetic sensitivity” OR “electromagnetic field sensitivity” OR EHS) AND TS = (well-being OR illness OR ill-health OR symptom* OR health OR health complaint* OR headache*) AND TS = (exposure OR provocation OR mobile phone* OR cell phone* OR visual display unit* OR powerline* OR base station* OR GSM OR UMTS OR TETRA OR electromagnetic OR electric OR electrical OR magnetic OR radiofrequency)

COCHRANE
http://onlinelibrary.wiley.com/cochranelibrary/search
(“electromagnetic hypersensitivity” OR electrohypersensitivity OR electrosensibility OR IEI-EMF OR “idiopathic environmental intolerance” OR “environmental intolerance” OR electrosensitivity OR electrosensitive OR “electric sensitivity” OR “Electrical sensitivity” OR “electromagnetic sensitivity” OR “electromagnetic field sensitivity” OR EHS) AND (well-being OR illness OR ill-health OR symptom* OR health OR health complaint* OR headache*) AND (exposure OR provocation OR mobile phone* OR cell phone* OR visual display unit* OR powerline* OR base station* OR GSM OR UMTS OR TETRA OR electromagnetic OR electric OR electrical OR magnetic OR radiofrequency)

PsychInfo
http://psycnet.apa.org/index.cfm?fa=search.defaultSearchForm
“electromagnetic hypersensitivity” OR electrohypersensitivity OR electrosensibility OR IEI-EMF OR “idiopathic environmental intolerance” OR “environmental intolerance” OR electrosensitivity OR electrosensitive OR “electric sensitivity” OR “Electrical sensitivity” OR “electromagnetic sensitivity” OR “electromagnetic field sensitivity”

EMF Portal
https://www.emf-portal.org/de/article/search/results?keywords=%22electromagnetic+hypersensitivity%22&logicalOperator=0&authors=&journals=&years=&topics%5B0%5D=0&frequencyRanges%5B0%5D=0&frequencyRanges%5B1%5D=1&frequencyRanges%5B2%5D=2&frequencyRanges%5B3%5D=3&frequencyRanges%5B4%5D=4&timeSpan=0&pageIndex=0

Rating tool for the evaluation of the methodological quality of individual studies

Table A.1: Criteria for reaching judgments on 16 key questions. The key questions are grouped into six domains for risk of bias and one domain for imprecision. Methodological alternatives marked in red are considered a source of high risk of bias, alternatives marked in yellow are considered a source of imprecision. (+) high risk of bias in favour of an effect of exposure, (-) high risk of bias in favour of a null result, (±) high risk of bias with uncertain direction on study outcome

SELECTION BIAS	
**Were individuals excluded whose EMF-attributed symptoms may be explained by somatic diseases or mental disorders?**	• Based on examination for somatic diseases<br>*Health status checked explicitly by a medical expert to prescreen individuals for study participation, exclusion of participants with acute or chronic somatic health problems that may explain the EMF-attributed symptoms*<br>• Based on examination for mental disorders<br>*Mental state checked explicitly by a psychologist/psychiatrist to prescreen individuals for study participation, exclusion of participants with psychiatric disorders that may explain the EMF-attributed symptoms or that may influence the individual's ability to give adequate responses in the experimental session*<br>• Based on medical interview<br>*Somatic diseases and mental disorders were queried in a (telephone) interview by e.g., trained study nurses to prescreen individuals for study participation, exclusion of participants with acute or chronic medical/mental conditions that may explain the EMF-attributed symptoms*<br>• Based on self-report of medical conditions<br>*Somatic diseases and mental disorders were queried in a questionnaire (e.g., Eltiti questionnaire*), exclusion of participants with acute or chronic medical/mental conditions and/or under medication that may explain the EMF-attributed symptoms*<br>• Not sufficiently considered/not reported (-)<br>*None of the alternatives specified above were reported. Insufficient if health status checked before the experiments but not used as an exclusion criterion; symptoms that participants reported may have been unrelated to EMF exposures*<br>**Eltiti S, Wallace D, Zougkou K, Russo R, Joseph S, Rasor P, et al. Development and evaluation of the electromagnetic hypersensitivity questionnaire. Bioelectromagnetics. 2007;28(2):137–51.*
**Was the contrast in the severity of symptoms between situations with/without exposure verified?**	• Based on open provocation for individual IEI-EMF participants<br>*Open provocation test to select individuals for the main experiment who showed a clear contrast in symptom development between active (exposure) and inactive conditions (sham)*<br>• Based on blinded pre-tests<br>*Blinded pre-test(s) to select individuals for following phase(s)/main tests, who showed a clear contrast in symptom development between active (exposure) and inactive conditions (sham)*<br>• Based on self-report<br>*Individuals were asked about frequency or severity of symptoms experienced when being exposed and when not being exposed or about the contrast between these situations (interview or questionnaire), inclusion of participants with a clear contrast in symptom development between the presence and absence of EMF*

	• Not reported (-)<br>*None of the alternatives specified above were reported; symptoms occurring due to other reasons than EMF exposure may have masked potential symptoms caused by EMF exposure*
**Were EMF exposures (type of exposure source, frequency range and exposure level) applied that individuals associate with their symptoms?**	• Based on open provocation<br>*Open provocation test (a) to select individuals for the main experiment, who developed symptoms when exposed to the applied type of exposure (e.g., mobile phone) or (b) to tailor the type of exposure according to the participant's reactions during open provocations (i.e., different exposures were tested and only exposure(s) to which the individual reacted was (were) applied in the main experiment for that individual)*<br>• Based on blinded pre-tests<br>*Blinded pre-test(s) (a) to select individuals for following phase(s)/ main tests who developed symptoms when exposed to the applied type of exposure or (b) to tailor the type of exposure according to the participant's reactions during the blinded pre-tests (i.e., different exposures were tested and only exposure(s) to which the individual reacted was (were) applied in the later phase(s)/the main tests for that individual)*<br>• Based on self-report<br>*Individuals were asked about EMF sources to which they attribute their symptoms (interview or questionnaire), (a) inclusion of those who reported being sensitive to the applied type of exposure or (b) tailoring of the exposure type according to the participant's self-report*<br>• Not reported (-)<br>*None of the alternatives specified above were reported; exposure types may have been unrelated to the symptoms*
**Were exposure durations and assessment times applied that matched the time scales for the symptoms to appear?**	• Based on open provocation<br>*Open provocation test (a) to select individuals for the main experiment for whom the exposure duration and assessment period were sufficiently long for the symptoms to develop and be registered or (b) to tailor the exposure duration and assessment times according to the participant's reactions during the open provocation test*<br>• Based on blinded pre-tests<br>*Blinded pre-tests (a) to select individuals for following phase(s)/ main tests for whom the exposure duration and assessment period were sufficiently long for the symptoms to develop and be registered or (b) to tailor the exposure duration and assessment times according to the participant's reactions during the blinded pre-tests*<br>• Based on self-report<br>*Individuals were asked about the time scales for the symptoms to appear (interview or questionnaire), (a) inclusion of those who reported time scales that fit to the experimental exposure duration and assessment times or (b) tailoring of the exposure duration and assessment times according to the participants' self-report*<br>• Not reported (-)<br>*None of the alternatives specified above were reported; the applied exposure durations and assessment periods may not have been sufficiently long for the symptoms to appear and to be registered*
**Were the intervals between exposure sessions sufficiently long to allow for recovery and to avoid carry-over effects?**	• Based on open provocation<br>*Open provocation test to (a) select individuals whose symptoms had disappeared within the applied interval or (b) to tailor the intervals between consecutive exposure sessions according to the participants' reactions following the open provocation test*

	• Based on self-report of usual recovery times or next exposure session delayed until all symptoms had vanished<br>*Individuals were asked about the time scales for the symptoms to disappear (interview or questionnaire), (a) inclusion of those who reported time scales that fit to the interval between exposure sessions (b) the interval between consecutive exposure sessions was adjusted to the recovery time of individual participants (either based on pre-reported recovery times or intervals were adjusted during the experimental sessions)*<br>• Interval of at least 1 week between exposure sessions<br>*Consecutive exposure sessions were separated by one week or more to presumably allow for full recovery of the participants and to presumably prevent carry-over effect*<br>• Interval of at least 1 day between exposure sessions<br>*Consecutive exposure sessions were separated by a period of 1-6 days*<br>• Not reported (±)<br>*None of the alternatives specified above were reported; reaction in one session may influence the reaction in the following session(s)*
**Were the symptoms recorded in the trials matched with those experienced in everyday exposure situations?**	• Based on symptoms reported in open provocation<br>*Open provocation test to register symptoms that participants developed during exposure, the same symptoms were queried during or after the blinded session(s)*<br>• Based on self-reported symptoms<br>*Individuals were asked about the symptoms (interview or questionnaire) that they usually develop when exposed to EMF sources, the same symptoms were queried during or after the blinded session(s)*<br>• Using a comprehensive list of symptoms for registration in the sessions or possibility to report any symptom<br>*Participants were asked to indicate the symptoms they had developed during or after the experimental session using a comprehensive list of symptoms that generally have been associated with EMF exposure or participants could report any symptom they had developed*<br>• Not reported (-)<br>*None of the alternatives specified above were reported; the queried symptoms may not have matched the symptoms that the individuals experience in everyday life*
**PERFORMANCE BIAS**	
**Was the level and method of blinding appropriate?**	• Blinding of participants during sessions*<br>*The participants were not aware of the exposure status during the experimental session*<br>• Blinding of research personal during sessions*<br>*The experimenter or the person who was in contact with the participants was not aware of the exposure status during the experimental session*<br>• Blinding of research personal during data analysis<br>*Information about the exposure status for each experimental trial was masked from the experimenter until after completion of data analysis*<br>• Removal of any clues that could reveal exposure status and/or tests done to control blinding<br>*Ensured by shielding, placement or modifications of equipment or by other means to prevent visual, audible, vibro-tactile, warmth sensations or other clues about the exposure condition and/or by testing the effectiveness of blinding*

	• No blinding of research personal during sessions (+)<br>*The experimenter or the person who was in contact with the participants was aware of the exposure status during the experimental session; unconscious or conscious signals to the participants might have influenced the response of participants*<br>• Insufficient removal of clues that could reveal exposure status and no tests done to control blinding (+)<br>*No information provided indicating that all clues that could reveal the exposure status were removed or that tests were done to confirm blinding, some clues might have influenced outcomes*<br>** The information that the experiments were conducted in a double-blind fashion, was rated as blinding of participants and blinding of research personal during sessions*
**Were biases related to sequence and period of the exposure conditions minimized (for studies with cross-over design)?**	• Randomized exposure sequence<br>*Randomization was based on a method with a random component*<br>• Counterbalanced exposure sequence<br>*Effects related to the order of exposures/shams were avoided*<br>• Use of a habituation session<br>*A training session, during which participants could become familiar with the laboratory environment, the experimental procedures and tests to reduce the effect of period, preceded the main experimental sessions; open provocation sessions are also considered a training session*<br>• Effect of sequence tested and/or controlled for in analysis (relevant if not counterbalanced)<br>*Possible effects related to the order of exposures/shams were considered and, if required, adjusted for in the data analysis*<br>• Same sequence and period of the exposure conditions for all participants or for all participants of a group (±)<br>*The sequence of the exposure conditions was the same for all participants or the period for any of the conditions was the same for all participants, which makes it impossible to differentiate between sequence and period effects versus effects of exposure*<br>• Not reported (±)<br>*No information was provided suggesting that the exposure sequence was randomized and that there was no significant deviation from counterbalance or control for effects of sequence and period; there may be sequence or period effects*
**CONFOUNDING BIAS**	
**Were biases related to confounders and cofactors minimized (for studies comparing parallel groups of IEI-EMF participants with different exposure conditions)?**	• Randomized allocation to EMF exposure or sham<br>*Each participant had an equal chance of being allocated to either EMF exposure or to sham or to one of different exposure scenarios, the allocation was adequately concealed*<br>• Confounding adjusted for in analysis<br>*Potential confounding factors and cofactors such as age, gender or self-rated hypersensitivity to EMF were considered and, if required, adjusted for in the data analysis*<br>• Not randomized (±)<br>*No information was provided suggesting that the allocation to the different exposure scenarios was randomized; no randomization may have resulted in participants with a higher probability in developing symptoms (irrespectively of reason) allocated to one experimental condition than to other conditions*

	• N/A<br>*For studies with cross-over design*
**Were other co-variates appropriately controlled?**	• Use of an adaptation period<br>*The experimental sessions were preceded by an adjustment/resting period (>10 minutes)*<br>• Sessions scheduled for the same time of day<br>*a) the time of day for different exposure scenarios (e.g., real and sham exposures) deviated less than 3 hours (e.g., all in the morning or all in the afternoon) to reduce effects that may result from variations in the diurnal rhythm of participants or (b) potential effects of sessions conducted at a different time of day were considered in the analysis*<br>• Inclusion of pre-trial symptom levels in analysis<br>*Baseline symptom levels were recorded before the experimental session and included in the analysis of symptom levels recorded during or after exposures*<br>• Control for pre-trial EMF exposure<br>*(a) Participants were instructed not to use devices with exposure similar to those that served as exposure source in the experiments (VDUs, mobile phones etc.) during a certain period of time before the experimental session or (b) such exposures were queried and potential effects considered in the analysis*<br>• Control for intake of drugs<br>*(a) Participants were instructed not to use drugs during a certain period of time before the experimental session or (b) drug intake was queried before the experimental session and potential effects were considered in the analysis*<br>• Control for relevant physical environment of the exposure room, e.g. humidity, light, temperature<br>*(a) All relevant parameters were controlled and kept constant throughout the experimental session or (b) the parameters were recorded and potential effects considered in the analysis*<br>• Refrain from e.g. caffeine and alcohol consumption, cigarettes, stress, strenuous exercise<br>*(a) Participants were instructed to avoid any factors that may influence the results during a certain period of time before the experimental session or (b) these factors were queried before the experimental session and potential effects considered in the analysis*<br>• Control for other potential sources of bias<br>*Other factors that vary between experimental sessions*<br>• None considered<br>*No information was provided on the control for potential confounders, there may have been other factors than the EMF exposure of importance for the symptoms*
**EXPOSURE BIAS**	
**Was the background exposure level controlled and minimized?**	• Based on provided background field levels or effectiveness of the shielding of the exposure room<br>*Measurement of field levels from ambient electric, magnetic and/or electromagnetic fields, testing rooms shielded or unshielded or located in places far away from power lines, base stations etc.*<br>• Use of shielded/rewired room or remote locations without providing exposure/shielding data<br>*Attenuation of electric, magnetic and electromagnetic fields from ambient*

	sources through e.g. absorbers and shielding precautions or testing room located in places far away from power lines, base stations etc. but without measurement of background field levels<br>• Reduction of exposure-unrelated EMF<br>*Ensured through e.g. removal/unplugging of other electrical devices or housing of the test equipment in an adjacent room*<br>• Based on open provocation with sham condition<br>*Background fields were low enough when they did not trigger symptoms during the sham condition*<br>• Not reported (-)<br>*None of the alternatives specified above were reported; participants may have been exposed to EMF exposure other than the exposure tested in the trials*
**Was the exposure level controlled?**	• Control of emission level from source<br>*By controlling the input power, using field/power meters and/or by regular calibration of the exposure system*<br>• Recording or estimation of exposure level<br>*Measurement or calculation of specific absorption rates (SAR) and/or incident power density and/or electric or magnetic fields in the area where the participants were seated*<br>• Not reported (-)<br>*None of the alternatives specified above were reported; participants may have been exposed to levels other than the provided levels*
**ATTRITION BIAS**	
**Were biases minimized that are related to attrition and to incomplete data included in the analysis?**	• No dropout or exclusion of participants or low dropout/exclusion rate<br>*No or very few losses of participants during the study or no indication that the loss is substantially differential (i.e., related to exposure conditions applied before the loss).*<br>• All data included in analysis for the reported outcomes or few missing outcome data<br>*Complete outcome data or few missing outcome data due to e.g. technical problems, outliers, errors or other reasons for loosing or excluding data or slight modifications of the study protocol which may not have biased the results to an appreciable extent*<br>• High attrition/exclusion rate or incomplete data in analysis (±)<br>*High loss or substantial differential loss of participants during the study and/or incomplete outcome data; responses of lost participants may have differed from responses of completing participants and missing data may have differed from included data*
**SELECTIVE REPORTING BIAS**	
**Was bias related to selective outcome reporting minimized?**	• All relevant outcomes reported<br>*All measured outcomes as outlined in the introduction and methods have been reported*<br>• Selective outcome reporting (±)<br>*One or several measured outcomes as outlined in the introduction or methods have not been reported, i.e., outcomes related to any types of symptoms, symptom scores, symptom levels; findings of not reported outcomes may have differed from those of reported outcomes*
**IMPRECISION**	

**Was the statistical power sufficient to detect participants whose symptoms are caused by a physical effect of EMF exposure?**	• Analysis based on individual data with sufficient number of repetitions to ensure statistical power<br>*A statistical power analysis was provided to calculate the minimum required number of repetitions for each condition, data analysis was done separately for each individual which would allow to identify individual, even one or a few, participants that suffer from IEI-EMF*<br>• Statistics based on group data and with sufficient number of participants/trials to ensure statistical power<br>*A statistical power analysis was provided to calculate the minimum required sample size and data analysis was done across participants which would allow to identify genuine effects*<br>• Analysis based on individual data for repeated trials or on group data without demonstration of sufficient statistical power<br>*No statistical power calculation was provided to calculate the minimum required number of repetitions for each condition/sample size, data analysis was done separately for individuals or across participants but it remained unclear whether the analysis allowed to identify genuine effects*<br>• Descriptive statistics only<br>*Quantitative description of the results without application of a statistical hypothesis test and this was not sufficient to draw conclusions about statistical significance*
**Were measures applied to control for the increased chance for false positive findings due to multiple comparisons?**	• By adjusting for multiple comparisons<br>*Adjusting p-values or significance level to account for the increased chance to reach significance due to multiple testing when conducting more than two statistical tests (also for studies with a pre-defined primary effect variable, i.e., one main symptom while other symptoms were secondary or explorative, and when performing statistics individually for a number of participants)*<br>• By retesting individuals with positive findings<br>*Verification of statistically significant results through retesting individual participants with a positive finding in the primary experiment, using the same experimental conditions*<br>• Not reported<br>*Adjustment for multiple comparisons as specified above not reported, or in studies performed at the individual level, retesting with positive findings not reported.*<br>• N/A<br>*In case of no multiple comparison, i.e., for studies which conducted not more than two statistical tests (e.g., examined one or two symptoms or analyses were based on a total symptom score) or in case of studies which did not provide a statistical analysis*

Methodological quality of individual studies

Table A.2: Rating of 16 key questions for individual studies. Key questions are grouped into six domains for risk of bias and one domain for imprecision. X: Applied method completely fulfilled the requirements to reduce the likelihood for false positive or false negative results; (x): applied method partly fulfilled the requirements to reduce the likelihood for false positive or false negative results. Red: Method considered a source of high risk of bias, yellow: method considered a source of imprecision

		VDU						ELF						RF															Various	
**Key question**	**Method**	1. Andersson et al. 1996	2. Flodin et al. 2000	3. Lonne-Rahm et al. 2000	4. Oftedal et al. 1995	5. Oftedal et al. 1999	6. Swanbeck & Bleeker 1989	7. Kim et al. 2012	8. Mc Carty 2011	9. Szemersky et al. 2105	10. Toomingas 1996	11. Trimmel & Schweiger 1998	12. Wenzel et al. 2005	13. Augner et al. 2009	14. Barth et al. 2000	15. Eltiti et al. 2007	16. Furubayashi et al. 2009	17. Hietanen et al. 2002	18. Hillert et al. 2008	19. Kwon et al. 2012	20. Nam et al. 2009	21. Nieto-Hernandez et al. 2011	22. Oftedal et al. 2007	23. Regel et al. 2006	24. Rubin et al. 2006	25. Verrender et al. 2017	26. Wallace et al. 2010	27. Wilén et al. 2006	28. Rea et al. 1991	*Number of studies*
**SELECTION BIAS**																														
**Were individuals excluded whose EMF-attributed symptoms may be explained by somatic diseases or mental disorders?**	based on examination for somatic diseases				X										X														(x)	2/1
	based on examination for mental disorders																													0
	based on medical interview	X		X	X																									3
	based on self-report of medical conditions		(x)		X			X		X						X	X		X	(x)	(x)	X	X	X	X	X	(x)	X		12/4
	not sufficiently considered/not reported					X	X		X		X	X	X	X				X												8

**Was the contrast in the severity of symptoms between situations with/without exposure verified?**	based on open provocation																									x				1
	based on blinded pre-tests																												x	1
	based on self-report	x	x		x	x	(x)		x						x								x							7/ 1
	not reported			x				x		x	x	x	x	x		x	x	x	x	x	x	x		x	x		x	x		18
**Were EMF exposures (type of exposure source, frequency range and exposure level) applied that individuals associate with their symptoms?**	based on open provocation			x											x			x					x			x				5
	based on blinded pre-tests																												x	1
	based on self-report	x	(x)	x	x	x	x	x	(x)	(x)					x	(x)	(x)	x	x	x	x	x	x	(x)	x	x	(x)	x		16 /7
	not reported										x	x	x	x																4
**Were exposure durations and assessment times applied that matched the time scales for the symptoms to appear?**	based on open provocation			x											x			x					x			x				5
	based on blinded pre-tests																												x	1
	based on self-report	x	x		x	x																x	x		x	x				8
	not reported						x	x	x	x	x	x	x	x		x	x		x	x	x			x			x	x		16
**Were the intervals between exposure sessions sufficiently long to allow for recovery and to avoid carry-over effects?**	based on open provocation																													0
	based on self-report of usual recovery times or next exposure session delayed until all symptoms had vanished		x			x			x									x				x			x	(x)				6/ 1
	interval of at least 1 week between exposure sessions	x		x	x	x		x								x			x					x			x			9
	interval of at least 1 day between exposure sessions		x				x													x	x	x	x		x			x		8
	not reported									x	x	x	x	x	x		x												x	8

**Were the symptoms recorded in the trials matched with those experienced in everyday exposure situations?**	based on symptoms reported in open provocation																					X			X				2
	based on self-reported symptoms	X		X	X	X		(x)							X				X	(x)		X	X		X	X				10 /2
	using a comprehensive list of symptoms for registration in the sessions or possibility to report any symptom		(x)		X		X		X	X	X		X			X		X									X	X	X	11 /1
	not reported											X		X			X				X			X						5

PERFORMANCE BIAS

Study number		1	2	3	4	5	6	7	8	9	10	11	12	13	14	15	16	17	18	19	20	21	22	23	24	25	26	27	28	
**Was the level and method of blinding appropriate?**	blinding of participants during sessions	X	X	X	X	X	X	X	X	X	X	X	X	X	X	X	X	X	X	X	X	X	X	X	X	X	X	X	X	28
	blinding of research personal during sessions	X	X	X	X	X	X	X	X	X		X	X	X	X	X	X	X	X	X		X	X	X	X	X	X		(x)	24 /1
	blinding of research personal during data analysis																													0
	removal of any clues that could reveal exposure status and/or tests done to control blinding	X	X	X	X	X		X	X	X	X			X	X	X	X	X	X	X	(x)	X	X	X	X	X		X		22 /1
	no blinding of research personal during sessions										X										X							X		3
	insufficient removal of clues that could reveal exposure status and no tests done to control blinding						X					X	X																X	.4
**Were biases related to sequence and period of the exposure conditions minimized (for studies with cross-**	randomized exposure sequence		X		X	X	X	X	X	X	X				X	X	X	(x)	X	X	X	X	X	X	X	X	X	X	X	22 /1
	counterbalanced exposure sequence	X					X	X									X			X	X	X	X		X	X	X			11
	use of a habituation session			X	X	X									X			X	X				X	X		X				9
	effect of sequence				X	X										X			X					X						5

		1	2	3	4	5	6	7	8	9	10	11	12	13	14	15	16	17	18	19	20	21	22	23	24	25	26	27	28	
**over design)?**	tested and/or controlled for in analysis (relevant if not counterbalanced)																													
	same sequence and period of the exposure conditions for all participants or for all participants of a group																													0
	not reported	X	X	X					X	X	X	X	X		X			X										X	X	12
	N/A													X																1

CONFOUNDING BIAS

Study number		1	2	3	4	5	6	7	8	9	10	11	12	13	14	15	16	17	18	19	20	21	22	23	24	25	26	27	28	
**Were biases related to confounders and cofactors minimized (for studies comparing parallel groups of IEI-EMF participants with different exposure conditions)?**	randomized allocation to EMF exposure or sham													X																1
	confounding adjusted for in analysis													X																1
	not randomized											X																		1
	N/A	X	X	X	X	X	X	X	X	X	X		X		X	X	X	X	X	X	X	X	X	X	X	X	X	X	X	26
**Were other co-variates appropriately controlled?**	use of an adaptation period	X						X					X				X		X	X	X	X	X		X			X		11
	sessions scheduled for the same time of day	X			X	X		X	N/A	N/A	N/A	N/A	N/A	N/A	N/A	X		N/A	X	X	X		X	X	X		X	X	N/A	13
	inclusion of pre-trial symptom levels in analysis	X	X	X			X	X						X			X		X	X	X	X	X	(x)	X	X	X			15 /1
	control for pre-trial EMF exposure						X												X				X	X						4
	control for intake of drugs																					X	X		X	X	X			5
	control for relevant factors of the physical environment of the exposure room, e.g., humidity, light,		(x)		X	X	X	X				X	X				(x)		(x)	X	X		(x)	X						9/ 4

	temperature																													
	refrain from e.g. caffeine and alcohol consumption, cigarettes, stress, strenuous exercise							X											X	X		X	X	X	X					7
	control for other potential sources of bias		X		X	X																							X	4
	none considered								X	X	X				X			X												5

EXPOSURE BIAS

Study number		1	2	3	4	5	6	7	8	9	10	11	12	13	14	15	16	17	18	19	20	21	22	23	24	25	26	27	28	
**Was the background exposure level controlled and minimized?**	based on provided background field levels or effectiveness of the shielding of the exposure room	X	X		X	X	(x)	X	X	X		(x)		X		X		X	X	X	X		X	X			X	(x)	(x)	16 /4
	use of shielded/ rewired room or remote location without providing exposure/shielding data			X													X													2
	reduction of exposure-unrelated EMF	X	X	X			(x)	X		X					X			X	X	X	X		X	X		X	X		X	15 /1
	based on open provocation with sham condition			X												X										X	X			4
	not reported										X		X									X			X					4
**Was the exposure level controlled?**	control of emission level from source									(x)						X	X	(x)		X	X	X	X	X		X		X		9/ 2
	recording or estimation of exposure level	X	X	X	X	X	X	(x)	X	X	(x)	(x)	(x)	X		X	X	X	X	X	X	X	X	X	X	X	X	X		22 /4
	not reported														X														X	2

ATTRITION BIAS

Study number		1	2	3	4	5	6	7	8	9	10	11	12	13	14	15	16	17	18	19	20	21	22	23	24	25	26	27	28	
**Were biases minimized that are related to attrition**	no dropout or exclusion of participants or low dropout/exclusion rate	X	X	X	X	X	X	X	X	X	X	X	X	X	X	X	X	X	X	X	X	X	X	X	X	X	X	X	X	28
	all data included in	X	X	X	X	X	X	X	X	X	X	X	X	X	X	X	X	X	X	X	X	X	X	X	X	X	X	X	X	28

**and to incomplete data included in the analysis?**	analysis for the reported outcomes or few missing outcome data																													
	high attrition/exclusion rate or incomplete data in analysis																													0
**SELECTIVE REPORTING BIAS**																														
Study number		1	2	3	4	5	6	7	8	9	10	11	12	13	14	15	16	17	18	19	20	21	22	23	24	25	26	27	28	
**Was bias related to selective outcome reporting minimized?**	all relevant outcomes reported	X		X	X	X	X	X	X	X	X	X	X	X		X	X	X	X	X	X	X	X	X	X	X	X	X	X	26
	selective outcome reporting		X												X															2
**IMPRECISION**																														
Study number		1	2	3	4	5	6	7	8	9	10	11	12	13	14	15	16	17	18	19	20	21	22	23	24	25	26	27	28	
**Was the statistical power sufficient to detect participants whose symptoms are caused by a physical effect of EMF exposure?**	analysis based on individual data with sufficient number of repetitions to ensure statistical power																									X				1
	statistics based on group data and with sufficient number of participants/trials to ensure statistical power									(x)						(x)						X	X		(x)		(x)			2/ 4
	analysis based on individual data for repeated trials or on group data without demonstration of sufficient statistical power	X	X	X	X	X		X	X			X		X	X		X		X	X	X			X				X		16
	descriptive statistics only						X				X		X					X											X	5
**Were measures applied to control for the increased**	by adjusting for multiple comparisons															X						X					X			3
	by retesting individuals with positive findings																												X	1

**chance for false positive findings due to multiple comparisons?**	not considered/not reported		X	X	X	X		X						X			X		X	X	X		X		X					12
	N/A	X					X		X	X	X	X	X		X			X						X		X		X		12
