# Supplementary material for: Methodological limitations in experimental studies on symptom development in individuals with idiopathic environmental intolerance attributed to electromagnetic fields (IEI-EMF) – a systematic review
Source: Environ Health. 2019 Oct 22;18:88. doi: 10.1186/s12940-019-0519-x (PMC6805477; doi:10.1186/s12940-019-0519-x)
Supplement: Supplementary file 2 — Aggregated ratings of 16 key questions. The key questions are grouped under six domains for risk of bias and one domain for imprecision. Included are the ratings for 28 studies. Some studies applied two or more methods to address a key question; they are therefore included more than once in the rating of a single key question. (PDF 252 kb) [file 12940_2019_519_MOESM2_ESM.pdf]

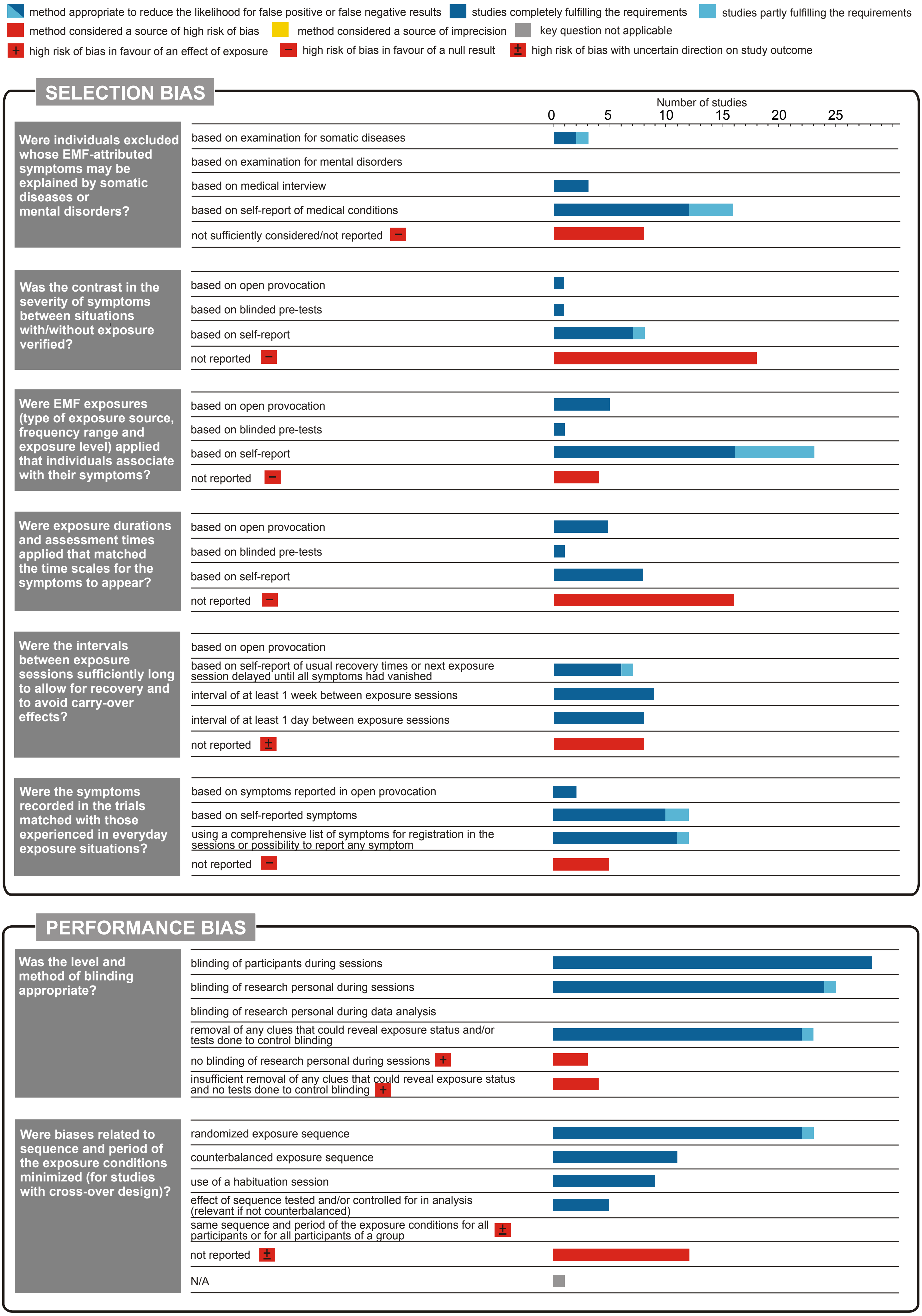

method appropriate to reduce the likelihood for false positive or false negative results
studies completely fulfilling the requirements
studies partly fulfilling the requirements
method considered a source of high risk of bias
method considered a source of imprecision
key question not applicable
+ high risk of bias in favour of an effect of exposure
− high risk of bias in favour of a null result
± high risk of bias with uncertain direction on study outcome
SELECTION BIAS
Number of studies
0
5
10
15
20
25
Were individuals excluded whose EMF-attributed symptoms may be explained by somatic diseases or mental disorders?
based on examination for somatic diseases
based on examination for mental disorders
based on medical interview
based on self-report of medical conditions
not sufficiently considered/not reported −
Was the contrast in the severity of symptoms between situations with/without exposure verified?
based on open provocation
based on blinded pre-tests
based on self-report
not reported −
Were EMF exposures (type of exposure source, frequency range and exposure level) applied that individuals associate with their symptoms?
based on open provocation
based on blinded pre-tests
based on self-report
not reported −
Were exposure durations and assessment times applied that matched the time scales for the symptoms to appear?
based on open provocation
based on blinded pre-tests
based on self-report
not reported −
Were the intervals between exposure sessions sufficiently long to allow for recovery and to avoid carry-over effects?
based on open provocation
based on self-report of usual recovery times or next exposure session delayed until all symptoms had vanished
interval of at least 1 week between exposure sessions
interval of at least 1 day between exposure sessions
not reported ±
Were the symptoms recorded in the trials matched with those experienced in everyday exposure situations?
based on symptoms reported in open provocation
based on self-reported symptoms
using a comprehensive list of symptoms for registration in the sessions or possibility to report any symptom
not reported −
PERFORMANCE BIAS
Was the level and method of blinding appropriate?
blinding of participants during sessions
blinding of research personal during sessions
blinding of research personal during data analysis
removal of any clues that could reveal exposure status and/or tests done to control blinding
no blinding of research personal during sessions +
insufficient removal of any clues that could reveal exposure status and no tests done to control blinding +
Were biases related to sequence and period of the exposure conditions minimized (for studies with cross-over design)?
randomized exposure sequence
counterbalanced exposure sequence
use of a habituation session
effect of sequence tested and/or controlled for in analysis (relevant if not counterbalanced)
same sequence and period of the exposure conditions for all participants or for all participants of a group ±
not reported ±
N/A

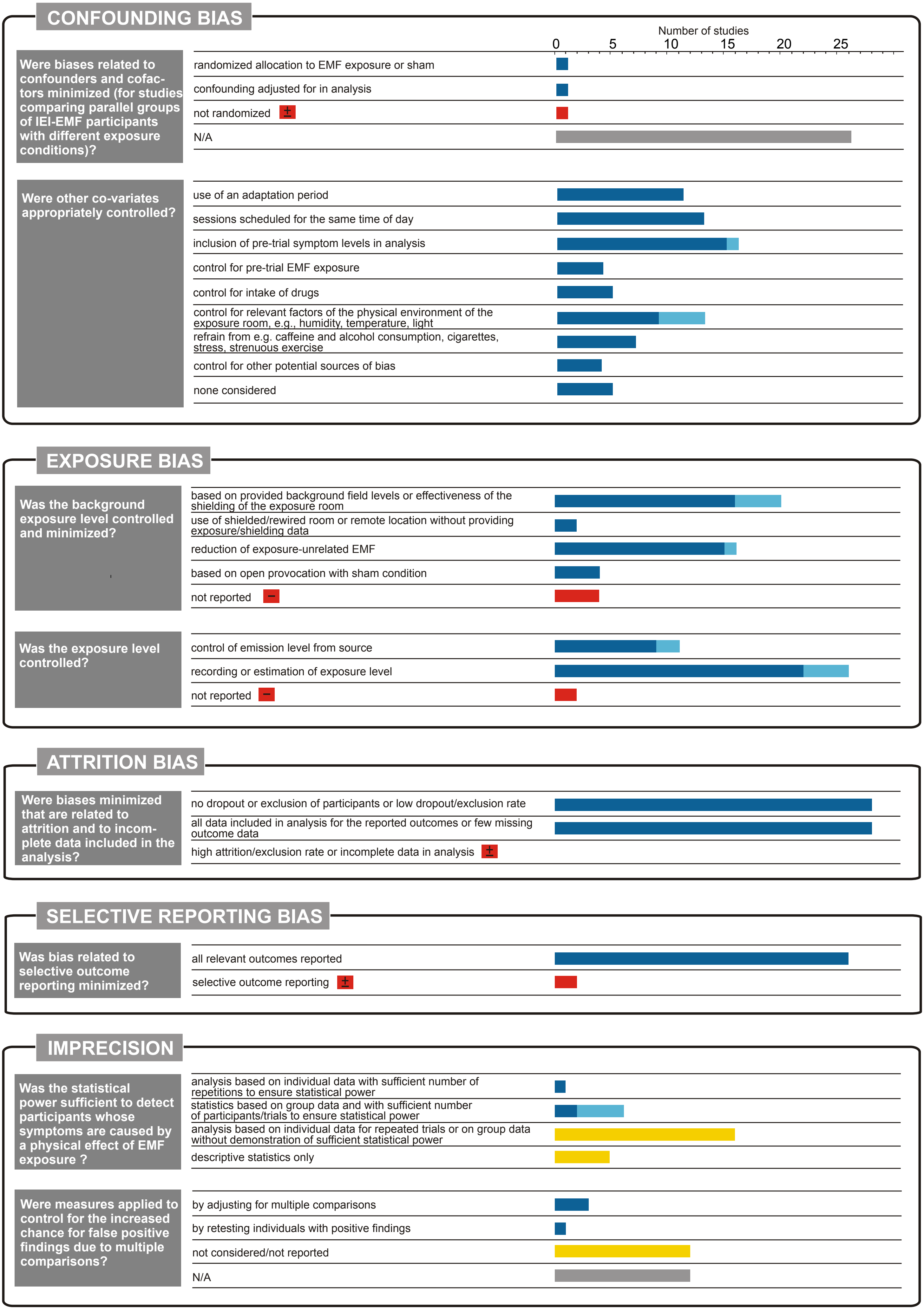
CONFOUNDING BIAS
Number of studies
0
5
10
15
20
25
Were biases related to confounders and cofactors minimized (for studies comparing parallel groups of IEI-EMF participants with different exposure conditions)?
randomized allocation to EMF exposure or sham
confounding adjusted for in analysis
not randomized ±
N/A
Were other co-variates appropriately controlled?
use of an adaptation period
sessions scheduled for the same time of day
inclusion of pre-trial symptom levels in analysis
control for pre-trial EMF exposure
control for intake of drugs
control for relevant factors of the physical environment of the exposure room, e.g., humidity, temperature, light
refrain from e.g. caffeine and alcohol consumption, cigarettes, stress, strenuous exercise
control for other potential sources of bias
none considered
EXPOSURE BIAS
Was the background exposure level controlled and minimized?
based on provided background field levels or effectiveness of the shielding of the exposure room
use of shielded/rewired room or remote location without providing exposure/shielding data
reduction of exposure-unrelated EMF
based on open provocation with sham condition
not reported −
Was the exposure level controlled?
control of emission level from source
recording or estimation of exposure level
not reported −
ATTRITION BIAS
Were biases minimized that are related to attrition and to incomplete data included in the analysis?
no dropout or exclusion of participants or low dropout/exclusion rate
all data included in analysis for the reported outcomes or few missing outcome data
high attrition/exclusion rate or incomplete data in analysis ±
SELECTIVE REPORTING BIAS
Was bias related to selective outcome reporting minimized?
all relevant outcomes reported
selective outcome reporting ±
IMPRECISION
Was the statistical power sufficient to detect participants whose symptoms are caused by a physical effect of EMF exposure ?
analysis based on individual data with sufficient number of repetitions to ensure statistical power
statistics based on group data and with sufficient number of participants/trials to ensure statistical power
analysis based on individual data for repeated trials or on group data without demonstration of sufficient statistical power
descriptive statistics only
Were measures applied to control for the increased chance for false positive findings due to multiple comparisons?
by adjusting for multiple comparisons
by retesting individuals with positive findings
not considered/not reported
N/A
